# 6-Mercaptopurine attenuates tumor necrosis factor-α production in microglia through Nur77-mediated transrepression and PI3K/Akt/mTOR signaling-mediated translational regulation

**DOI:** 10.1186/s12974-016-0543-5

**Published:** 2016-04-13

**Authors:** Hsin-Yi Huang, Hui-Fen Chang, Ming-Jen Tsai, Jhih-Si Chen, Mei-Jen Wang

**Affiliations:** Department of Medical Research, Buddhist Tzu Chi General Hospital, Hualien, Taiwan; Department of Emergency Medicine, Ditmanson Medical Foundation Chiayi Christian Hospital, Chiayi, Taiwan

**Keywords:** 6-Mercaptopurine, Microglia, TNF-α, Nur77, Nuclear factor-κB, Histone H3 acetylation, PI3K/Akt, mTOR

## Abstract

**Background:**

The pathogenesis of several neurodegenerative diseases often involves the microglial activation and associated inflammatory processes. Activated microglia release pro-inflammatory factors that may be neurotoxic. 6-Mercaptopurine (6-MP) is a well-established immunosuppressive drug. Common understanding of their immunosuppressive properties is largely limited to peripheral immune cells. However, the effect of 6-MP in the central nervous system, especially in microglia in the context of neuroinflammation is, as yet, unclear. Tumor necrosis factor-α (TNF-α) is a key cytokine of the immune system that initiates and promotes neuroinflammation. The present study aimed to investigate the effect of 6-MP on TNF-α production by microglia to discern the molecular mechanisms of this modulation.

**Methods:**

Lipopolysaccharide (LPS) was used to induce an inflammatory response in cultured primary microglia or murine BV-2 microglial cells. Released TNF-α was measured by enzyme-linked immunosorbent assay (ELISA). Gene expression was determined by real-time reverse transcription polymerase chain reaction (RT-PCR). Signaling molecules were analyzed by western blotting, and activation of NF-κB was measured by ELISA-based DNA binding analysis and luciferase reporter assay. Chromatin immunoprecipitation (ChIP) analysis was performed to examine NF-κB p65 and coactivator p300 enrichments and histone modifications at the endogenous TNF-α promoter.

**Results:**

Treatment of LPS-activated microglia with 6-MP significantly attenuated TNF-α production. In 6-MP pretreated microglia, LPS-induced MAPK signaling, IκB-α degradation, NF-κB p65 nuclear translocation, and in vitro p65 DNA binding activity were not impaired. However, 6-MP suppressed transactivation activity of NF-κB and TNF-α promoter by inhibiting phosphorylation and acetylation of p65 on Ser276 and Lys310, respectively. ChIP analyses revealed that 6-MP dampened LPS-induced histone H3 acetylation of chromatin surrounding the TNF-α promoter, ultimately leading to a decrease in p65/coactivator-mediated transcription of TNF-α gene. Furthermore, 6-MP enhanced orphan nuclear receptor Nur77 expression. Using RNA interference approach, we further demonstrated that Nur77 upregulation contribute to 6-MP-mediated inhibitory effect on TNF-α production. Additionally, 6-MP also impeded TNF-α mRNA translation through prevention of LPS-activated PI3K/Akt/mTOR signaling cascades.

**Conclusions:**

These results suggest that 6-MP might have a therapeutic potential in neuroinflammation-related neurodegenerative disorders through downregulation of microglia-mediated inflammatory processes.

**Electronic supplementary material:**

The online version of this article (doi:10.1186/s12974-016-0543-5) contains supplementary material, which is available to authorized users.

## Background

Brain inflammation is a typical feature of neurodegenerative diseases and acute forms of brain injury. Numerous in vivo clinical imaging and neuropathology studies suggest that activated microglia, the resident immunocompetent and phagocytic cells in the central nervous system (CNS), play a critical role in the pathogenesis of neurodegenerative disorders, including Alzheimer’s disease (AD), Parkinson’s disease (PD), and multiple sclerosis [1–3]. Under physiological conditions, microglia are involved in immune surveillance and host defense against infectious agents. However, microglia readily become activated in response to neuronal injury or immunological challenges. Although microglial activation is an indispensable defense mechanism against pathogens, uncontrolled and overactivated microglia can trigger neurotoxicity. Release of pro-inflammatory and cytotoxic factors, such as interleukin-1β, tumor necrosis factor-α (TNF-α), nitric oxide (NO), and reactive oxygen species (ROS) are believed to contribute to the neurotoxic effects caused by activated microglia [3]. Therefore, control of inflammation in the brain, involving a host of cytokines induced by microglia, might be important for regulation of numerous pathological processes of these diseases.

TNF-α is a pro-inflammatory cytokine that is upregulated in the brain in response to various insults or injury. TNF-α is released predominantly by activated microglia and may contribute to primary or secondary tissue injury [4, 5]. Within the brain, inflammatory processes might be modulated by TNF-α through further activation of microglia and astrocytes [6]. TNF-α is known to induce changes in mitochondrial ultrastructure and function, and it also induces ROS and NO generation thus further promoting the inflammatory response and exacerbating the neuronal damage [7, 8]. In addition, TNF-α can directly induce neuronal death by binding to TNF receptor 1 to trigger intracellular death-related signaling pathways [9]. TNF-α has been implicated as an important factor for the onset and perpetuation of neurodegenerative diseases, since increased levels of this cytokine are present in the affected areas in many neurodegenerative diseases [10–12]. Several lines of evidence support the concept that excess TNF-α plays a central role in AD [10, 13]. Application of a biologic TNF-α inhibitor significantly improves symptoms of AD patients [14]. Furthermore, the cerebrospinal fluid and postmortem brains of PD patients display elevated levels of TNF-α and its receptors [11]. Using engineered dominant-negative TNF variants and the decoy TNF receptor to block soluble TNF signaling demonstrating that TNF-dependent mechanisms are required for loss of dopaminergic neurons in models of PD [15]. In amyotrophic lateral sclerosis (ALS), both TNF-α and soluble TNF receptor levels are raised in serum of patients [12]. Administration of a TNF-α antagonist has been shown to extend lifespan and slow motor dysfunction in a mouse model of ALS [16]. Therefore, TNF-α is a key cytokine of the immune system that initiates and promotes neuroinflammation, which under uncontrolled conditions may lead to the development of neurodegenerative diseases.

6-Mercaptopurine (6-MP) belongs to the thiopurines, a group of substances structurally related to endogeneous purine bases like adenine, guanine, and hypoxanthine. 6-MP and its long-lived prodrug, azathioprine, are among the oldest pharmacological immunosuppressive agents in use today. 6-MP has been used for the treatment of acute childhood leukemia and chronic myelocytic leukemia, inflammatory bowel disease, systemic lupus erythematosus, inflammatory myopathies, and rheumatoid arthritis [17, 18]. It has also been used for the prevention of acute rejection in organ transplant patients [17]. 6-MP is converted to 6-thioguanine nucleotides that act as purine analogs and incorporated into newly synthesized DNA, which has long been considered to be the proposed therapeutic mechanism [19]. More work now has expanded the function of this drug by demonstrating that 6-MP can target biological activities outside of the purine biosynthesis pathway including regulation of Bcl-2/Bax ratio [20], Rac1-mediated signaling [21–23], and transcriptional activity of orphan nuclear receptor NR4A family members [24, 25]. Moreover, 6-MP inhibits atherosclerosis in a mouse model through decreasing lesion monocyte chemoattractant chemokine-1 (MCP-1) levels and reducing macrophage content [26]. Recently, Chang et al. [27] demonstrated that 6-MP exerts a neuroprotective effect on permanent focal cerebral occlusion in rats. However, little is known about the effects of 6-MP in the CNS, especially in microglia in the context of neuroinflammation. In the current study, we investigated whether 6-MP downregulates microglial inflammatory responses through decreasing microglial TNF-α production and elucidated the possible mechanisms of this modulation.

## Methods

### Materials

Lipopolysaccharide (LPS) from *Escherichia coli* serotype O111:B4 and rapamycin were obtained from Calbiochem (San Diego, CA). 6-Mercaptopurine was from Sigma-Aldrich (St. Louis, MO). Cell culture ingredients were purchased from Invitrogen (Carlsbad, CA). Polyclonal rabbit anti-acetyl histone H3 (Ac-H3) was from Upstate Biotechnology (Lake Placid, NY). Monoclonal rabbit anti-Nur77, polyclonal rabbit anti-NOR-1, polyclonal rabbit anti-acetyl p65 (Lys310), and polyclonal rabbit anti-phospho-p65 (Ser276) were obtained from Abcam (Cambridge, MA). Polyclonal rabbit anti-Nurr1 and polyclonal rabbit anti-p300 was purchased from Santa Cruz Biotechnology (Santa Cruz, CA). All other antibodies were from Cell Signaling Technology (Beverly, MA). All other reagents were from Sigma-Aldrich (St. Louis, MO).

### Microglial cultures

Murine BV-2 microglial cells were maintained in Dulbecco’s modified Eagle’s medium (DMEM) supplemented with 10 % fetal bovine serum (FBS), 100 U/ml penicillin, and 100 μg/ml streptomycin at 37 °C in a humidified incubator under 5 % CO_2_. Confluent cultures were trypsanized. Cells were plated into 24-wells plate at a density of 1 × 10^5^ cells per well and then incubated for 24 h before treatment. Primary microglia were prepared from ventral mesencephalon of 1-day-old Sprague-Dawley rats as previously described [28]. Briefly, ventral mesencephalic tissues, devoid of meninges and blood vessels, were dissociated by a mild mechanical trituration. The isolated cells (5 × 10^7^) were seeded in 150-cm^2^ culture flasks in DMEM containing 10 % FBS, 100 U/ml penicillin, and 100 μg/ml streptomycin. The cells were maintained at 37 °C in a humidified atmosphere of 5 % CO_2_ and 95 % air. The medium were changed 4 days later. Upon reaching confluence (12–14 days), microglia were separated from astrocytes by shaking the flasks for 2 h at 180 rpm. Detached cells were plated into 24-wells at a density of 2.5 × 10^5^ cells per well. After 2 h of incubation at 37 °C, nonadherent cells were removed. The purity of microglia cultures was assessed by using OX-42 antibody, and more than 95 % of cells were stained positively. Cells were cultured for 2 days before treatment.

### Real-time RT-PCR analysis

The expression of TNF-α and Nur77 gene were quantified using real-time reverse transcription polymerase chain reaction (RT-PCR) analysis. Briefly, total RNA was extracted from microglia cultures with TRIzol® reagent (Invitrogen). One-step real-time RT-PCR analysis was performed to determine the expression of genes (Power SYBR® Green RNA-to-C_T_^TM^ 1-step kit, Applied Biosystems, Foster City, CA). The primer sequences are as follows: for mouse TNF-α, 5′-TTC TGT CTA CTG AAC TTC GGG GTG ATC GGT CC-3′ and 5′-GTA TGA GAT AGC AAA TCG GCT GAC GGT GTG GG-3′; for mouse Nur77, 5′-AGC TTG GGT GTT GAT GTT CC-3′ and 5′-AAT GCG ATT CTG CAG CTC TT-3′; for mouse Nurr1, 5′-TCA CCT CCG GTG AGT CTG ATC-3′ and 5′-TGC TGG ATA TGT TGG GTA TCA TCT-3′; for mouse NOR-1, 5′-CGC CGA AAC CGA TGT CA-3′ and 5′-TGT ACG CAC AAC TTC CTT AAC CA-3′; for mouse β-actin, 5′-GGC TGT ATT CCC CTC CAT CG-3′ and 5′-CCA GTT GGT AAC AAT GCC ATG T-3′; for rat TNF-α, 5′-CAG GGC AAT GAT CCC AAA GTA-3′ and 5′-GCA GTC AGA TCA TCT TCT CGA-3′; for rat Nur77, 5′-CCG GTG ACG TGC AGC AAT TTT ATG AC-3′ and 5′-GGC TAG AAT GTT GTC TAT CCA GTC ACC-3′; for rat β-actin, 5′-CAC CCG CGA GTA CAA CCT TC-3′ and 5′-CCC ATA CCC ACC ATC ACA CC-3′. Threshold cycle (C_t_) value for each test gene was normalized to the C_t_ value for the β-actin control from the same RNA preparations. The ratio of transcription of each gene was calculated as 2^-(**∆**Ct)^, where ∆C_t_ is the difference C_t (test gene)_ − C_t (β-actin)_.

### Western blotting

Microglial cells were lysed in M-PER® Mammalian Protein Extraction Reagent (Pierce, Rockford, IL). Protein concentration was determined by Bradford assay (Bio-Rad, Hercules, CA,); 30~50 μg of protein sample was separated on 10~12 % sodium dodecyl sulfate-polyacrylamide gel (SDS-PAGE) and transferred to immobilon polyvinylidene difluoride (PVDF) membranes (Merck Millipore, Billerica, MA). The membranes were incubated in Tris-buffered saline (TBST, 0.1 M Tris/HCl, pH 7.4, 0.9 % NaCl, 0.1 % Tween 20) supplemented with 5 % dry skim milk for 1 h to block nonspecific binding. After rinsing with TBST buffer, the membranes were incubated with primary antibodies against Nur77, Nurr1, and NOR-1; phosphorylated members of the MAP kinase family; the specific phosphorylated sites were IκB-α (Ser32), NF-κB p65 (Ser276, Ser468 and Ser536), Akt (Ser473), S6K (Thr389), 4E-BP1 (Ser64 and Thr69), vasodilator-stimulated phosphoprotein (VASP) (Ser157), and MSK1 (Ser376); and an acetylated site of NF-κB p65 (Lys310). Antibodies active against all forms of each mentioned protein, histone deacetylase 1 or β-actin, were used as internal controls to determine loading efficiency. The membranes were washed three times with TBST followed by incubation with appropriate horseradish peroxidase-conjugated secondary antibodies. The antigen-antibody complex was detected by using an ECL chemiluminescence detection system (PerkinElmer, Boston, MA). The intensity of the bands was quantified with a GS-800 calibrated densitometer (Bio-Rad) and calculated as the optical density × area of bands.

### DNA binding assay

Nuclear extracts were prepared by using the NE-PER® nuclear and cytoplasmic extraction reagents (Pierce) as per the manufacturer’s instructions. The DNA binding assay was performed as described [29] with some modifications. Briefly, 5~10 μg of nuclear extracts were mixed with poly dI-dC (50 μg/ml) in a binding buffer (10 mM HEPES, pH 7.9, 50 mM KCl, 0.5 mM EDTA, 0.5 mM DTT, 3 mM MgCl_2_, 5 % glycerol, 0.5 mg/ml BSA, 0.05 % NP-40) and then incubated in 96-well plates coated with immobilized biotin-labeled oligonucleotides (2 pmol per/well) for 1 h at room temperature. Following three washes, primary antibody specific to NF-κB p65 was added and incubated again at room temperature for 1 h. Addition of secondary antibody conjugated to horseradish peroxidase was performed prior to the quantification of NF-κB DNA-binding activity by measuring luminescence (FLx800 Fluorescence Reader, Biotek Instruments, Inc., Winooski, VT). The following biotin-labeled oligonucleotides were used: the consensus NF-κB, 5′-CACAGTTG- AGGGGACTTTCCCAGGC-3′; the four putative NF-κB sequences within the mouse TNF-α promoter, κB1 (−856): 5′-GGGGGAGGGGAATCCTTGGAAGAC-3′; κB2 (−659): 5′-GAG- GTCCGTGAATTCCCAGGGCTG-3′; κB3 (−512): 5′-CAAACAGGGGGCTTTCCCTCCT-CA-3′; κB4 (−214): 5′-GACGGGGAGGAGATTCCTTGATGC-3′.

### Transient transfection and luciferase assay

TNF-α promoter luciferase construct (TNF-α-Luc) was created by ligating a 1.2-kb *Kpn*I-*Xho*I fragment of the mouse TNF-α promoter into the pGL3 basic plasmid (Promega, Madison, WI). The fragment was amplified by PCR with the following primers: forward, 5′-CGGGGTACCGATTCTGTCTGCTTGTGTCT-3′ and reverse, 5′-CGGCTCGAGGTTCTGGAGTTTCTGTTCTC-3′. NF-κB promoter luciferase construct (NF-κB-Luc) was purchased from Clontech Laboratories (Palo Alto, CA) and contains four repeats of consensus NF-κB binding sequence. Each construct was transfected into BV-2 cells using TurboFect Transfection Reagent (Thermo Scientific, Lafayette, CO) following the manufacturer’s protocols. At 24 h after transfection, cells were treated with LPS in the absence or presence of 6-MP for another 6 h. Luciferase activity of cell lysates was determined luminometrically by the luciferase assay system (Promega) as specified by the manufacturer. Each transfection was performed in duplicate, and all experiments were repeated at least three times. Luciferase activity of each sample was normalized to the protein content of the extracts. Luciferase activity from the untreated sample was arbitrarily set at 1.0 for the calculation of fold induction.

### Chromatin immunoprecipitation

BV-2 cells were pretreated with 50 μM 6-MP for 16 h followed by stimulation with 100 ng/ml LPS for the indicated times. Cells were cross-linked with 1 % formaldehyde and stored at −80 °C before use. Chromatin immunoprecipitation (ChIP) assays were performed using SimpleChIP® Enzymatic Chromatin IP kit (Cell Signaling Technology, Beverly, MA) following the manufacturer’s protocols. Cross-linked chromatin was enzymatic digested to generate fragments with a length of approximately 150–900 bp (1 to 5 nucleosomes). The chromatin was subjected to immunoprecipitation using the following antibodies: anti-p65, anti-p300, anti-acetyl-histone H3, and normal rabbit IgG (Abcam). Immunoprecipitated DNA fragments were collected by Protein G magnetic beads. DNA/protein complexes were eluted from the beads and reverse cross-linked at 65 °C for 2 h in the presence of Proteinase K. Purified DNA were subjected to real-time PCR using primers specific to NF-κB binding site of mouse TNF-α promoter. The sequences of the primers used for ChIP and the PCR product size are as follows: κB1, 5′-GAG AAG TGA CTC CAC TGG AGG GT-3′ and 5′-ACT GCG GTA CAT CAA CTC AGA CAT-3′ (−912 to–763, 150 bp); κB2, 5′-AAG GCT TGT GAG GTC CGT GA -3′ and 5′-AAG TGG CTG AAG GCA GAG CA-3′ (−675 to–532, 144 bp); κB3, 5′-ATG CAC ACT TCC CAA CTC TCA AG-3′ and 5′-CTT CTG AAA GCT GGG TGC ATA AG-3′ (−575 to–457, 119 bp); and κB4, 5′-TCT GGA GGA CAG AGA AGA AAT G-3′ and 5′-GGT TTG GAA AGT TGG GGA CAC C-3′ (−353 to–172, 182 bp). The abundance of the immunoprecipitated DNA in a sample was normalized to the amount of signal in the input DNA. The values of the untreated cultures or LPS-treated alone cultures were set to 1.0 or 100 %, respectively.

### Immunoprecipitation

Following LPS stimulation, cells were then harvested with NE-PER® nuclear and cytoplasmic extraction reagents. The nuclear extracts (120 μg of protein) were incubated with a rabbit anti-Nur77 or a rabbit anti-p65 antibody with gentle rocking overnight at 4 °C. PureProteome^TM^ protein G magnetic beads (Merck Millipore) were added (15 μl of suspension) and rotated for 3 h at 4 °C. After washing the beads with ice-cold immunoprecipitation buffer (20 mM Tris, pH 7.5, 150 mM NaCl, 1 mM EDTA, 1 mM EGTA, 1 % Triton X-100, 2.5 mM sodium pyrophosphate, 1 mM β-glycerolphosphate, 1 mM Na_3_VO_4_, 1 μg/ml leupeptin, 1 mM PMSF), immunoprecipitated proteins were eluted in sample buffer and subjected to Western blotting analyses with anti-Nur77 and anti-P65 antibodies.

### RNA interference

Mouse On-TARGET plus® SMARTpool Nur77 siRNA were obtained from Dhmarcon (Thermo Scientific). Nonspecific siRNA was used as negative control. BV-2 cells were seeded in 24-well plates for 16 h prior to transfection. siRNA duplexes were transfected into BV-2 cells using Lipofectamine® RNAiMAX Transfection Reagent (Invitrogen) according to the protocol of the manufacturer. After 48-h transfection, BV-2 cells were transfected with a TNF-α promoter or 4XNF-κB-luciferase reporter construct followed by exposure to LPS with or without 6-MP pretreatment, and luciferase activity was assayed. For TNF-α mRNA expression, released TNF-α assay, or ChIP analysis, cells were pretreated with 50 μM 6-MP for 16 h prior to LPS exposure after siRNA transfection.

### TNF-α assay

Primary microglia and BV-2 microglia were stimulated with LPS in the absence or presence of 6-MP, and supernatants were collected and kept frozen in aliquots at −80 °C until use. Release of TNF-α was measured with a commercial enzyme-linked immunosorbent assay (ELISA) kit from R&D Systems (Minneapolis, MN) according to the manufacturer’s instructions.

### Statistical analysis

All data are expressed as mean ± SEM. Data were analyzed by one-way ANOVA followed by Scheffe’s test. For paired analyses, *t* test was used. A *p* value less than 0.05 was considered statistically significant.

## Results

### 6-MP decreases TNF-α production in LPS-stimulated microglia

**Fig. 1:**
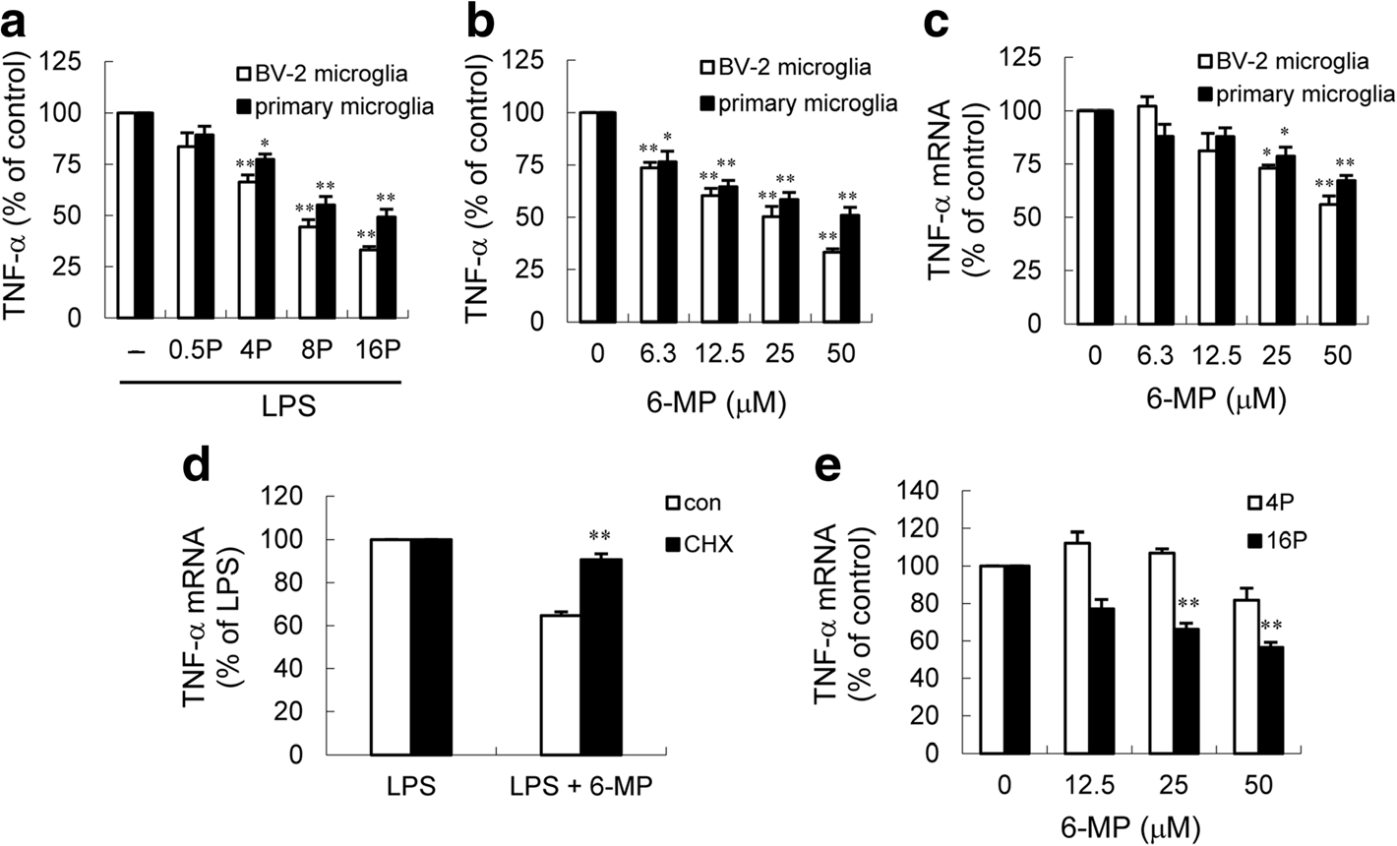
6-MP attenuates TNF-α production in microglia. **a** Cells were pretreated (*P*) with vehicle or 50 μM 6-MP for the indicated times, cells were then stimulated with 100 ng/ml LPS for 6 h. TNF-α secretion into the culture medium was analyzed by ELISA. The relative differences between control and 6-MP pretreated groups were calculated and expressed as percent (%) of control. Data are presented as mean ± SEM for three independent experiments. *p < 0.05; **p < 0.01 compared with control. **b**, **c** Cells were pretreated with vehicle or various concentrations of 6-MP for 16 h followed by treatment with 100 ng/ml LPS for 6 h (**b**) or 2 h (**c**). Released TNF-α (**b**) was measured by ELISA. Expression of TNF-α mRNA (**c**) was quantified by real-time RT-PCR as described in the “Methods” section. Data are presented as mean ± SEM for three independent experiments. *p < 0.05; **p < 0.01 compared with control. The TNF-α content in untreated cultures was not detectable. The levels of TNF-α in LPS-treated alone BV-2 cells and primary microglia were 16.0 ± 0.4 and 2.8 ± 0.3 ng/ml, respectively. **d** The suppression of TNF-α mRNA by 6-MP requires de novo protein synthesis. BV-2 cells were pretreated with cyclohexamide (CHX, 0.5 μg/ml) or its vehicle 1 h before 16-h incubation without or with 6-MP (50 μM) and subsequent LPS stimulation (100 ng/ml) for 2 h. TNF-α mRNA expression was analyzed by real-time RT-PCR. Data are presented as mean ± SEM for three independent experiments. **p < 0.01 compared with control. **e** BV-2 cells were pretreated (*P*) with various concentrations of 6-MP for 4 h or 16 h followed by exposure to 100 ng/ml LPS for 2 h. Total RNA was then extracted for real-time RT-PCR analysis. Data are presented as mean ± SEM for three independent experiments. **p < 0.01 compared with respective control

Because TNF-α is a key cytokine that initiates and promotes inflammation in the brain, we tested whether 6-MP might modulate microglial activation by examining the effect of 6-MP on LPS-induced TNF-α release in microglia. Microglia were pretreated with 6-MP for 0.5~16 h prior to stimulation with 100 ng/ml LPS. Interestingly, TNF-α protein levels were significantly reduced in cells pretreated with 6-MP for 4 h or longer but not for 0.5 h (Fig. 1a). Furthermore, Fig. 1b shows that LPS-induced TNF-α release was markedly suppressed by 6-MP in a dose-dependent manner. 6-MP has been shown to exert antiproliferative and cytotoxic effects resulting primarily from the inhibition of purine biosynthesis at multiple steps and incorporation into nucleic acids as thioguanine nucleotide [19]. To exclude the possibility that 6-MP caused cytotoxic effects which led to the decrease in TNF-α, MTT assay was used to evaluate the cell viability following treatment with 6-MP. We found that the concentrations used in this study did not show cytotoxic effects (data not shown). To investigate whether the reduction in TNF-α protein in microglia following 6-MP exposure is due to suppression of TNF-α mRNA expression, real-time RT-PCR analysis was performed to assess the TNF-α mRNA levels. The results showed that 6-MP pretreatment significantly reduced LPS-induced TNF-α mRNA expression in a dose-dependent manner (Fig. 1c). Such prolonged pretreatment required for 6-MP’s inhibitory effect suggests that the effect might be dependent on de novo protein synthesis. To test this hypothesis, the effect of cycloheximide, a protein synthesis inhibitor, on 6-MP-mediated inhibition of TNF-α mRNA expression was examined. Cycloheximide treatment was shown to reverse the suppressive effect of 6-MP on TNF-α mRNA expression (Fig. 1d), suggesting that a protein induced by the 6-MP signals likely suppressed TNF-α mRNA synthesis induced by LPS. Additionally, Fig. 1e shows that pretreatment of BV-2 cells with 12.5–50 μM 6-MP for 4 h did not markedly inhibit TNF-α mRNA expression, whereas the protein levels of TNF-α were significantly reduced in cultures pretreated with 50 μM (Fig. 1a). These data raise the possibility that 6-MP may also exert an ability to control TNF-α mRNA translation.

### 6-MP does not affect intracellular Toll-like receptor (TLR) signaling

**Fig. 2:**
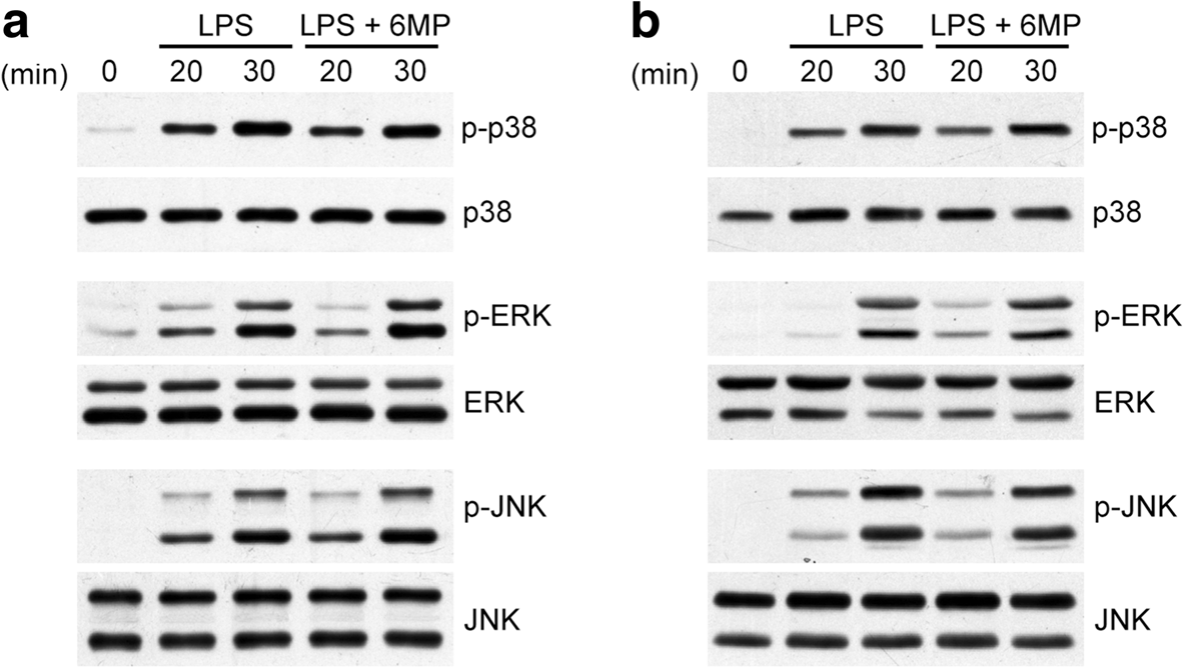
6-MP does not inhibit LPS-induced activation of MAPKs signals. BV-2 cells (**a**) and primary microglia (**b**) were pretreated with vehicle or 6-MP (50 μM) for 16 h and stimulated with LPS (100 ng/ml) for the indicated times. Whole cell extracts were prepared. Western analysis was used to determine LPS-induced p38, ERK, and JNK activation in the absence or presence of 6-MP. The immunoblots are representative of three independent experiments

**Fig. 3:**
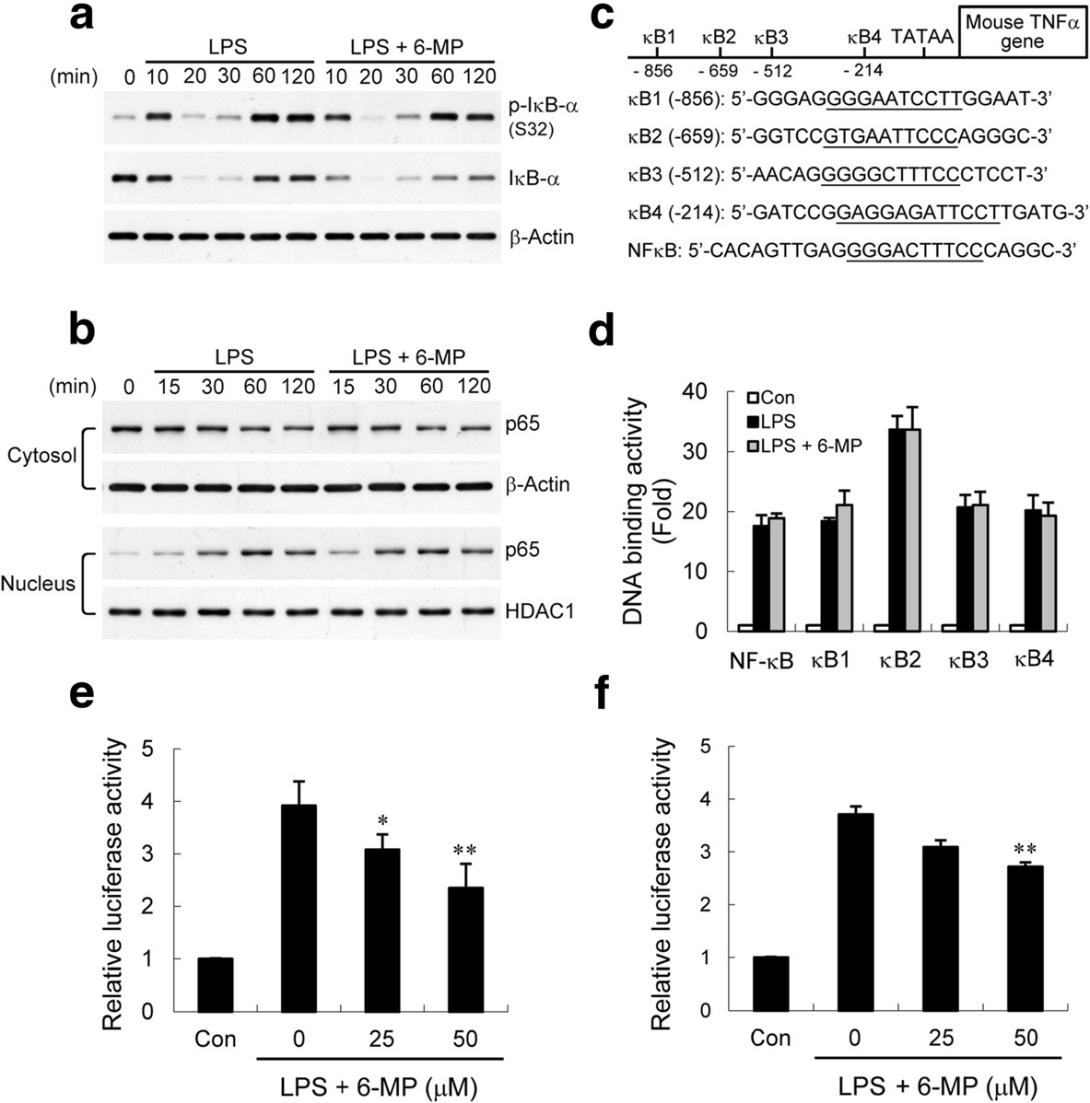
6-MP decreases LPS-induced transactivation of NF-κB and TNF-α promoter. BV-2 cells were preincubated with vehicle or 6-MP (50 μM) for 16 h before stimulation with 100 ng/ml LPS for the various times indicated. Whole cell lysates and nuclear extracts were prepared. **a** IκB-α degradation is not suppressed by 6-MP. Western analysis was used to determine total and phosphorylated IκB-α proteins in whole cell extracts. **b** Translocation of p65 from cytosol to the nucleus was determined by immunoblotting. β-Actin and histone deacetylase 1 (HDAC1) immunoblotting were performed to monitor loading for cytosol and nuclear proteins, respectively. **c** Putative 4-κB sites within the mouse TNF-α promoter (κB1 to 4) and a consensus κB sequence are schematically depicted. The *underlines* indicate the binding sequences for NF-κB. **d** Cells were pretreated with vehicle or 6-MP (50 μM) for 16 h before stimulation with 100 ng/ml LPS for 60 min. Nuclear proteins were isolated. ELISA-based measurement of p65 DNA binding was analyzed as described in the “Methods” section. **e**, **f** Cells were transfected with a 4XNF-κB (**e**) or TNF-α promoter (**f**) luciferase construct; 24 h post-transfection, cells were preincubated with vehicle or various concentrations of 6-MP for 16 h before stimulation with 100 ng/ml LPS for 6 h. Luciferase activity is presented as a fold of control. Data are presented as mean ± SEM for four independent experiments. *p < 0.05; **p < 0.01 compared with LPS alone

LPS induces the production of proinflammatory cytokines through intracellular phosphorylation signaling cascades activated by TLR4, including MAP kinase and NF-κB signaling cascade [30–32]. We next determined whether or not the de novo synthesis proteins induced by 6-MP exert their ability on suppression of TNF-α mRNA is by disrupting the TLR signaling. Activation of three MAPKs, including p38, ERK, and JNK, was analyzed by Western blotting. As shown in Fig. 2, 6-MP was not able to block LPS-induced phosphorylation of three MAPKs in microglia. Activation of the IκB kinase (IKK) complex depends on phosphorylation and has been demonstrated to be critical for NF-κB activation. After phosphorylation of IκB-α by the IKK complex, IκB-α is degraded and releases NF-κB [33]. Therefore, we determined the effect of 6-MP on IκB-α phosphorylation and degradation. 6-MP failed to suppress LPS-induced IκB-α phosphorylation and degradation (Fig. 3a), suggesting that the action of 6-MP may be mediated through impairment of the downstream signaling of IKK activation and IκB-α degradation. Upon TLR signaling, the transcription factor NF-κB is translocated into the nucleus and upregulated cytokine mRNA transcription. The nuclear localization of the p65 subunit of NF-κB was also unaffected by 6-MP pretreatment (Fig. 3b).

### 6-MP downregulates LPS-stimulated transcriptional activity of TNF-α promoter

TNF-α gene transcription is largely predicated by binding of NF-κB to its *cis* regulatory elements within the TNF-α promoter [34]. Four putative κB sites have been identified in the murine TNF-α promoter. These sites are termed κB1 to −4 and are located at the nucleotide positions −856, −659, −512, and −214, respectively ([35, 36], Accession No.: NC_000083.6). As mentioned above, 6-MP pretreatment did not appear to affect MAPK and NF-κB activation, we then tested a possibility that 6-MP might inhibit TNF-α transcription by directly interfering with NF-κB binding to its DNA element. To explore this possibility, nuclear proteins were extracted from BV-2 cells pretreated with 6-MP and stimulated with LPS. ELISA-based assay was performed to assess NF-κB p65 binding to four putative κB sequence located within the mouse TNF-α promoter (Fig. 3c) as well as to the consensus κB sequence. Following LPS stimulation, a marked increase in amount of p65 bound to all oligonucleotides tested (Fig. 3d). Pretreatment with 6-MP had no inhibitory effects on LPS-induced p65 DNA binding, indicating that 6-MP does not change the binding of p65 to the κB sites in an in vitro assay.

To further assess whether the suppressive effect of 6-MP on TNF-α gene transcription is mediated by downregulating TNF-α promoter activity, we transient transfection BV-2 cells with a TNF-α promoter-luciferase construct (TNF-α-Luc). Because TNF-α promoter activity is largely dependent on κB elements, we also assessed the effects of 6-MP on NF-κB promoter using a reporter gene with 4× κB sequences (NF-κB-Luc). LPS treatment alone prominently elicited transcriptional activity of the TNF-α and NF-κB consensus promoter, and this effect was significantly attenuated by concomitant 6-MP pretreatment (Fig. 3e, f). These findings confirm that the inhibitory effect of 6-MP on TNF-α gene expression is, at least partially, mediated by inhibition of NF-κB transactivation activity.

### 6-MP inhibits NF-κB p65 phosphorylation and acetylation

**Fig. 4:**
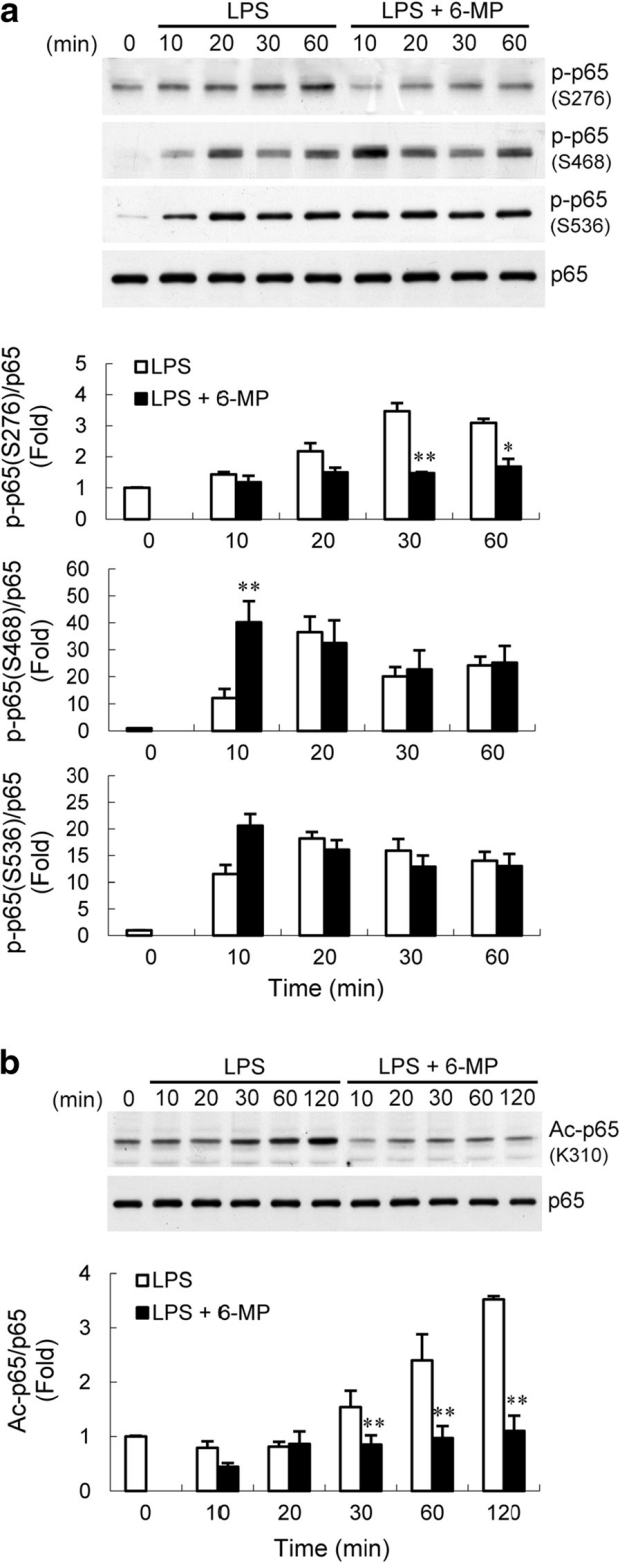
6-MP inhibits NF-κB p65 phosphorylation at Ser276 and acetylation at Lys310. BV-2 cells were pretreated with vehicle or 6-MP (50 μM) for 16 h and then stimulated with LPS (100 ng/ml) for the indicated times. Whole cell lysates were prepared and subjected to western blotting using antibodies specific for phosphorylated (Ser276, 468, and 536) (**a**), acetylated (Lys310) (**b**), or total forms of p65. Data are presented as mean ± SEM for three independent experiments. *p < 0.05; **p < 0.01 compared with respective cultures treated with LPS alone

NF-κB activation, characterized by phosphorylation of specific amino acid residues in the p65 subunit, is one important prerequisite for transactivation of the target genes [37–39]. Therefore, one possible mechanism by which 6-MP may control LPS-induced NF-κB activity may be through direct phosphorylation of NF-κB. We included in our analysis the assessment of phosphorylation of p65. In control BV-2 cells, LPS stimulation resulted in enhanced phosphorylation of p65 at Ser276, 468, and 536 (Fig. 4a). Cells pretreated with 6-MP showed a compromised induction of phosphorylation at Ser276 but not 468 or 536 upon treatment with LPS. Zhong et al. [40] have proposed that the catalytic subunit of PKA is maintained in an inactive state through direct interaction with IκB in the NF-κB/IκB complex. LPS-induced degradation of IκB-α releases the kinase to phosphorylate the vicinal substrate p65 at Ser276. To determine if this mechanism for phosphorylation of p65 is suppressed by 6-MP, we assessed the LPS-induced PKA activation by detecting the phosphorylation of VASP at Ser157, which is a typical surrogate of PKA activity [41]. The results showed that 6-MP had no inhibitory effect on LPS-induced VASP phosphorylation (Additional file 1: Figure S1). This is consistent with the observations that 6-MP did not inhibit IκB-α degradation induced by LPS (Fig. 3a). Furthermore, the nuclear kinase mitogen- and stress-activated protein kinase-1 (MSK1) is another candidate for phosphorylation of this site [42, 43]. To determine further whether 6-MP inhibits activation of MSK1, BV-2 cells were stimulated with LPS in the absence or presence of 6-MP pretreatment. Western blot analysis showed that LPS-provoked phosphorylation (activation) of MSK1 was not affected by 6-MP (Additional file 1: Figure S1). The data suggest that 6-MP’s inhibitory action on phosphorylation of p65 at Ser276 is not likely to be mediated through impairment of the PKA or MSK1 activation.

In addition, NF-κB signaling is also modulated by post-translational modifications, including reversible acetylation of the p65 subunit [44]. Full transcriptional activity of p65 requires acetylation of Lys310 [45]. Using an antibody specific for acetylated Lys310, we found that LPS induced increased levels of acetylated p65 (Fig. 4b). Treatment with 6-MP diminished levels of p65 with acetylated Lys310. These results suggest that 6-MP downregulates NF-κB activation, possibly by inhibiting phosphorylation and acetylation of p65 on Ser276 and Lys310, respectively.

### 6-MP attenuates recruitment of p65 and the coactivator p300 to the TNF-α promoter and modifies histone H3 acetylation

**Fig. 5:**
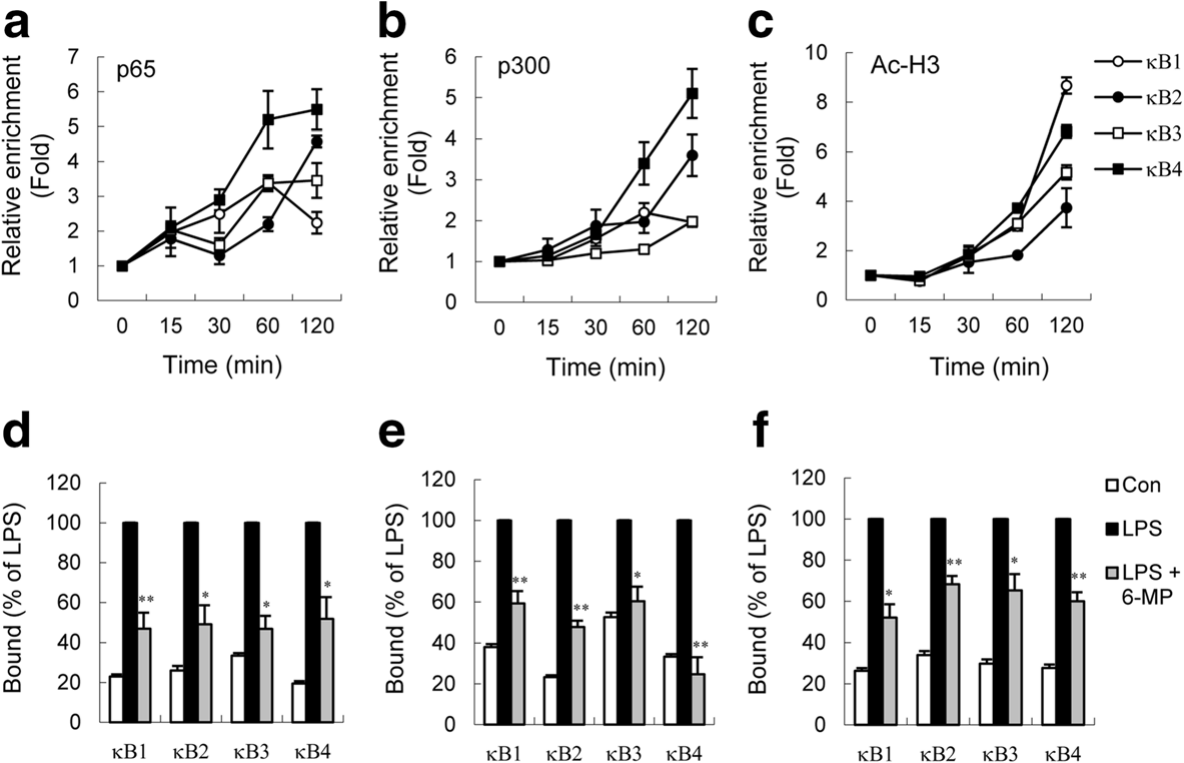
6-MP reduces LPS-induced p65 and coactivator p300 recruitment and histone H3 acetylation at the TNF-α promoter. **a–c** BV-2 cells were stimulated with 100 ng/ml LPS for the indicated times and chromatin immunoprecipitation (ChIP) assays were performed as described in the “Methods” section. The TNF-α promoter regions encompassing κB1-κB4 were targeted for ChIP analyses assessing associations of p65 (**a**), p300 (**b**), and acetylated histone H3 (Ac-H3) (**c**). The abundance of the immunoprecipitated DNA in a sample was normalized to the amount of signal in the input DNA. The values of the untreated samples were set to 1.0. Data are presented as mean ± SEM for three independent experiments. **d–f** BV-2 cells were pretreated with vehicle or 6-MP (50 μM) for 16 h followed by exposure to 100 ng/ml LPS for 60 min. ChIP analyses were carried out with anti-p65 (**d**), anti-p300 (**e**), and anti-Ac-H3 (**f**) antibodies. The values of the LPS alone-treated samples were set to 100 %. Data are presented as mean ± SEM for five independent experiments. *p < 0.05; **p < 0.01 compared with respective LPS alone

6-MP treatment was shown to reduce TNF-α mRNA expression and TNF-α promoter activity. To provide further support for the hypothesis that 6-MP modulates LPS-induced endogenous transcription of TNF-α, a ChIP assay was performed to confirm 6-MP action on the interactions of p65 with the mouse TNF-α promoter region. The ChIP assay revealed a time-dependent recruitment of the p65 subunit of NF-κB to the TNF-α promoter region of four putative κB sites after LPS stimulation (Fig. 5a). Moreover, the reduced LPS-induced occupancy of p65 at TNF-α promoter was observed in cultures pretreated with 6-MP (Fig. 5d). Regulation of NF-κB activity has also been shown to take place through its binding to transcriptional coactivators. Cyclic-AMP response element binding protein (CREB)-binding protein (CBP) and its homologue p300 are transcriptional coactivators known to interact with p65 [46, 47]. Additionally, histone acetylation of lysine residues requires the activities of histone acetyltransferases, such as CBP/p300, which are important in relaxing the compact structure of nucleosomes [48]. The effect of 6-MP on the recruitment of coactivator p300 to the TNF-α promoter and LPS-induced histone modifications were analyzed. p300 was weakly associated with the TNF-α promoter in untreated BV-2 cells, and LPS stimulation strongly promoted recruitment of p300 to the κB2 and κB4 sites at 30–120 min, whereas the occupancy of p300 at κB1 and κB3 was slightly increased (Fig. 5b). Similar to p65, 6-MP inhibited LPS-induced recruitment of p300 to the four κB sites of TNF-α promoter (Fig. 5e). In addition, in untreated cells, histone H3 was weakly acetylated on the TNF-α promoter, and acetylation of histone H3 was enhanced in a time-dependent manner following LPS exposure (Fig. 5c). Enrichment of Ac-H3 induced by LPS was also decreased in 6-MP-pretreated cells (Fig. 5f). These findings collectively suggest that 6-MP may keep promoter in a deacetylated state following treatment with LPS and reduce the association of p65-p300 complex with the promoter to inactivate the transcription of TNF-α gene.

### Nur77 is required for 6-MP-induced suppression of TNF-α production

**Fig. 6:**
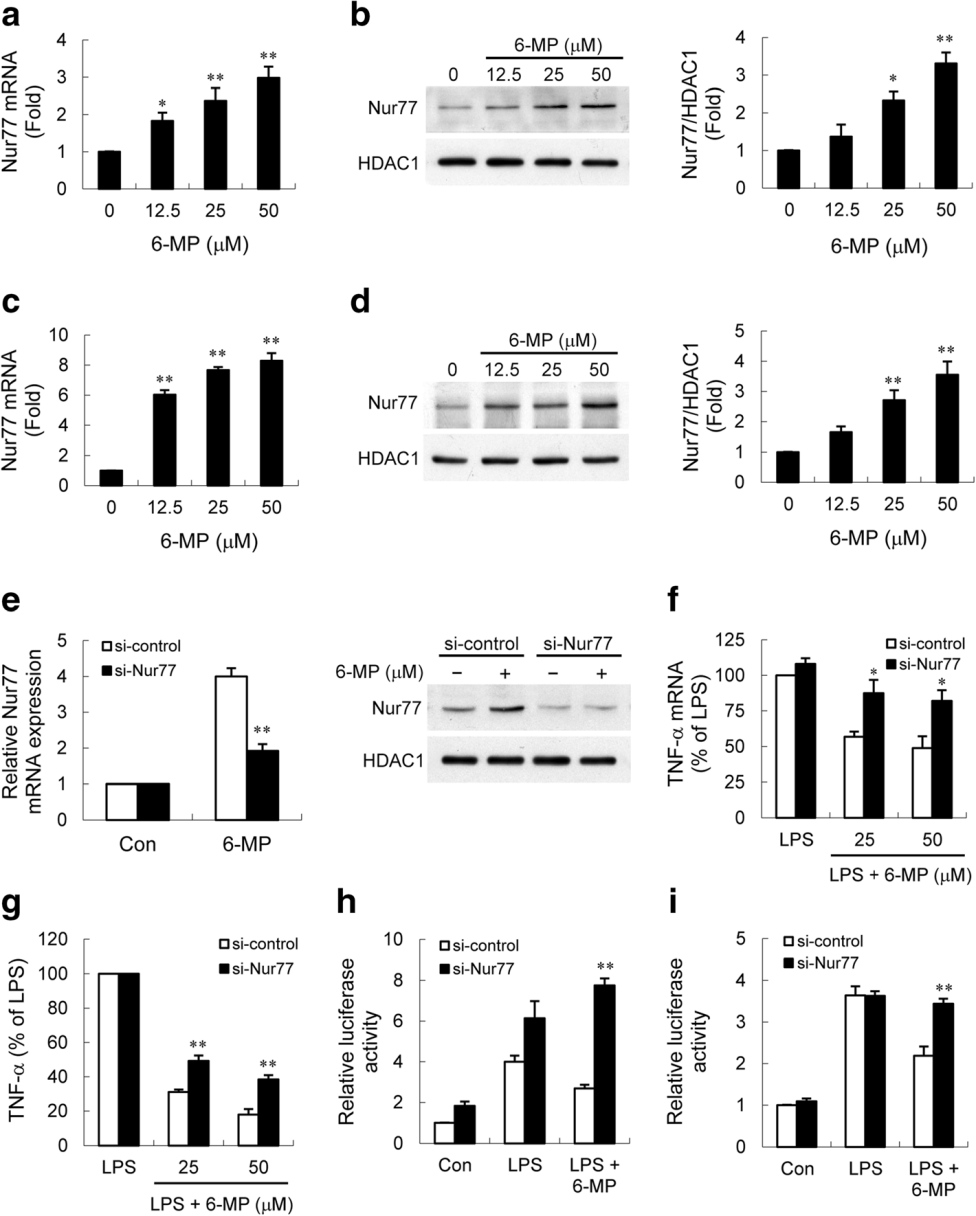
Upregulated Nur77 is involved in 6-MP-mediated inhibitory effect on TNF-α production. BV-2 cells (**a**, **b**) and primary microglia (**c**, **d**) were treated with vehicle or various concentrations of 6-MP for 1 h (**a**, **c**) or 16 h (**b**, **d**). The expression of Nur77 mRNA (**a**, **c**) was quantified by real-time RT-PCR. Nuclear extracts were prepared and subjected to western blotting (**b**, **d**) using antibody specific for Nur77. HDAC1 immunoblotting was performed to monitor loading. Data are presented as mean ± SEM for three independent experiments. *p < 0.05; **p < 0.01 compared with control. **e** BV-2 cells were transfected with control or Nur77 siRNA for 48 h followed by treatment with 6-MP (50 μM) for 1 h (*left*) or 16 h (*right*). Total RNA and nuclear proteins were extracted. Expression levels of Nur77 mRNA and protein were analyzed by real-time RT-PCR (*left*) and western blotting (*right*), respectively. **p < 0.01 compared with 6-MP-treated control siRNA transfected cells. **f**, **g** After siRNA transfection, BV-2 cells were pretreated with 25 and 50 μM 6-MP for 16 h followed by treatment with LPS for another 2 h (**f**) or 6 h (**g**). Analyses of TNF-α mRNA expression and released TNF-α were performed as described in Fig. 1. Data are presented as mean ± SEM for three independent experiments. LPS-induced TNF-α production in control siRNA and Nur77 siRNA transfected cells were 25.1 ± 2.4 and 27.0 ± 3.5 ng/ml, respectively. *p < 0.05; **p < 0.01 compared with 6-MP-pretreated control siRNA transfected cells. **h**, **i** Nur77 contributes to 6-MP-mediated inhibition of NF-κB and TNF-α promoter transcriptional activities. After siRNA transfection, NF-κB (**h**) or TNF-α promoter (**i**) luciferase construct was transfected into BV-2 cells for 24 h. The transfected cells were pretreated with 50 μM 6-MP for 16 h followed by treatment with LPS for another 6 h. Luciferase activity from the non-stimulated cells transfected with control siRNA was arbitrarily set at 1.0 for the calculation of fold. Data are presented as mean ± SEM for four independent experiments. **p < 0.01 compared with 6-MP-pretreated control siRNA transfected cells

Our investigation demonstrated that the inhibitory effects of 6-MP was shown to be dependent on de novo protein synthesis. Previous studies reported that 6-MP increases the transactivation of the NR4A orphan nuclear receptors in several cell types [24, 25, 49]. These receptors have been shown to resolve inflammation [50–53]. To determine whether 6-MP induces NR4A receptors expression in microglia, real-time RT-PCR analysis and western blotting were performed to assess the NR4A receptors mRNA and protein levels, respectively. The results showed that 6-MP upregulated Nur77 and Nurr1, but had no effect on NOR-1 mRNA expression in BV-2 cells (Fig. 6a, Additional file 2: Figure S2). In contrast to Nur77 (Fig. 6b), the change of Nurr1 protein level induced by 6-MP was not observed in our experiments (Additional file 2: Figure S2). These findings indicate that the protein levels of all three NR4A receptors are not simultaneously increased by 6-MP in BV-2 cells. 6-MP-elevated Nur77 expression was further validated in primary microglia (Fig. 6c, d). Nur77 has been reported to exert an anti-inflammatory property in various cell and animal models [52–56]. To substantiate the significance of 6-MP-induced Nur77 in microglial activation, we performed loss-of-function studies, using the RNA interference technique. Knockdown of Nur77 expression in 6-MP-pretreated BV-2 cells (Fig. 6e) led to a significant decrease in 6-MP-mediated inhibition of TNF-α expression (Fig. 6f, g). Furthermore, the inhibitory effect of 6-MP on LPS-induced NF-κB and TNF-α promoter activity was relieved in cells transfected with Nur77 siRNA (Fig. 6h, i). Thus, downregulation of Nur77 showed defective 6-MP-mediated suppression of LPS-induced TNF-α production, demonstrating that Nur77 contributes to 6-MP’s inhibitory effects.

**Fig. 7:**
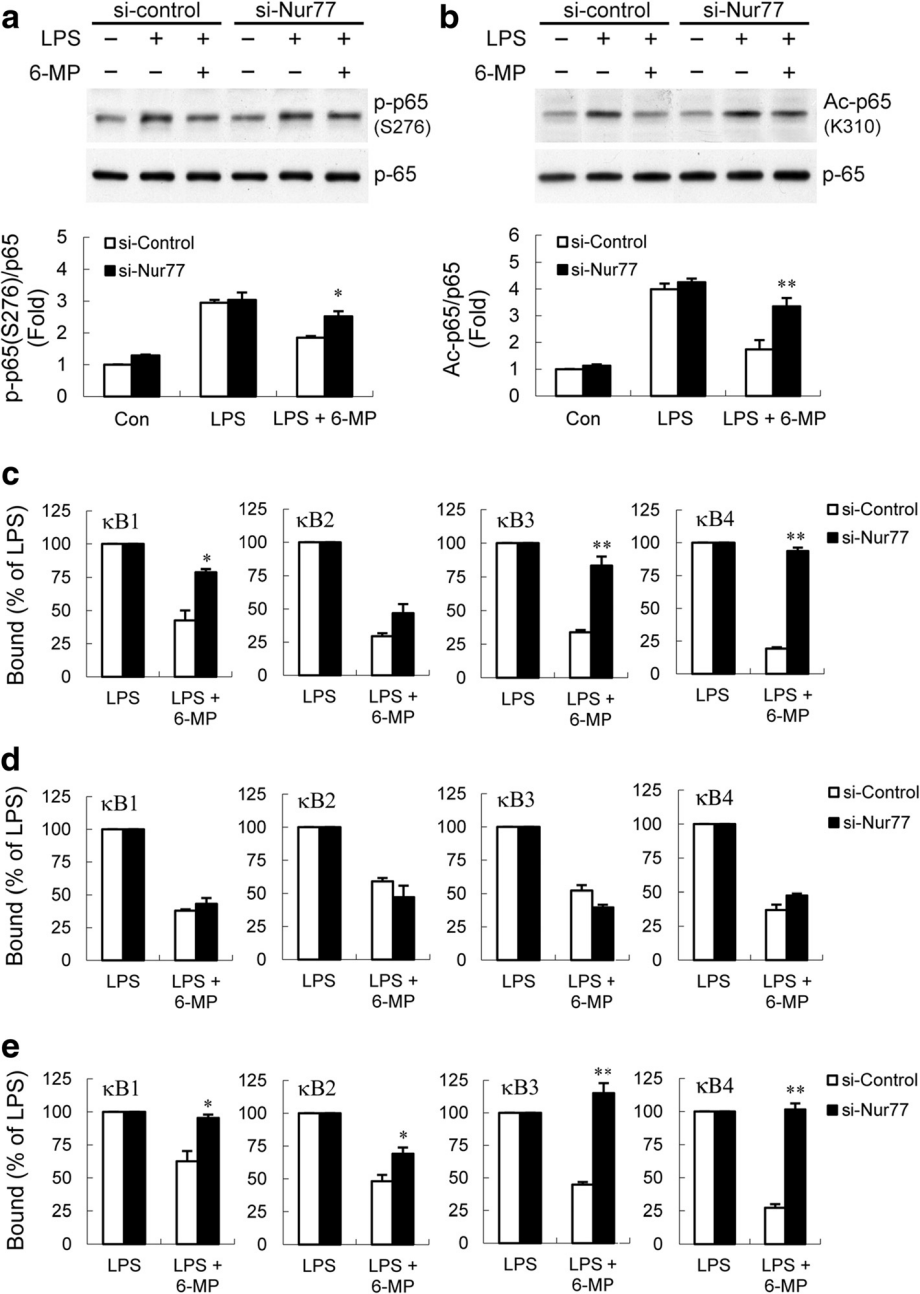
Nur77 is required for 6-MP-mediated inhibition of p65 post-translational modifications, p65 recruitment, and histone H3 acetylation. BV-2 cells were transfected with control or Nur77 siRNA for 48 h. Cells were pretreated with 50 μM 6-MP for 16 h followed by stimulation with 100 ng/ml LPS for 20 min (**a**) or 60 min (**b–e**). The levels of phosphorylated p65 (Ser276) (**a**) and acetylated p65 (Lys310) (**b**) were determined by western blotting. ChIP assays were performed with anti-p65 (**c**), anti-p300 (**d**), and anti-Ac-H3 (**e**) antibodies. The detection of the immunoprecipitated TNF-α promoter was analyzed by PCR with promoter-specific primers. *p < 0.05; **p < 0.01 compared with 6-MP-pretreated control siRNA transfected cells

It has been shown that Nur77 attenuates p65 transcriptional activity by directly interacting with p65 to weaken its binding to DNA [57, 58]. Therefore, the physical protein-protein interaction between Nur77 and p65 was examined. Following LPS stimulation, Nur77 and p65 showed interaction in immunoprecipitation (IP) assays and the amount of p65 (or Nur77) that precipitated with Nur77 (or p65) was enhanced by 6-MP (Additional file 3: Figure S3). The results of IP assays are not consistent with the fact that 6-MP has no influence on p65 binding to DNA, suggesting that the increased Nur77-p65 interaction by 6-MP might be not yet sufficient to directly block the binding of p65 to its response element. To further investigate whether Nur77 is responsible for the 6-MP’s actions on inhibition of p65 post-translational modifications, we knock down the expression of Nur77 by using specific small interfering RNA. As shown in Fig. 7a, b, 6-MP-mediated inhibition of p65 phosphorylation at Ser276 and acetylation at Lys310 were reversed after knockdown of Nur77. Furthermore, ChIP assays demonstrated that downregulation of Nur77 could restore 6-MP-mediated reduction of p65, but not p300, recruitment and histone H3 acetylation at TNF-α promoter regions (Fig. 7c–e). Our results provide evidence for 6-MP impact on NF-κB pathway, likely via Nur77-mediated inhibition of p65 phosphorylation and acetylation, and an epigenetic regulation mechanism.

### 6-MP blocks LPS-induced TNF-α production by inhibiting PI3-kinase/Akt/mTOR signaling

**Fig. 8:**
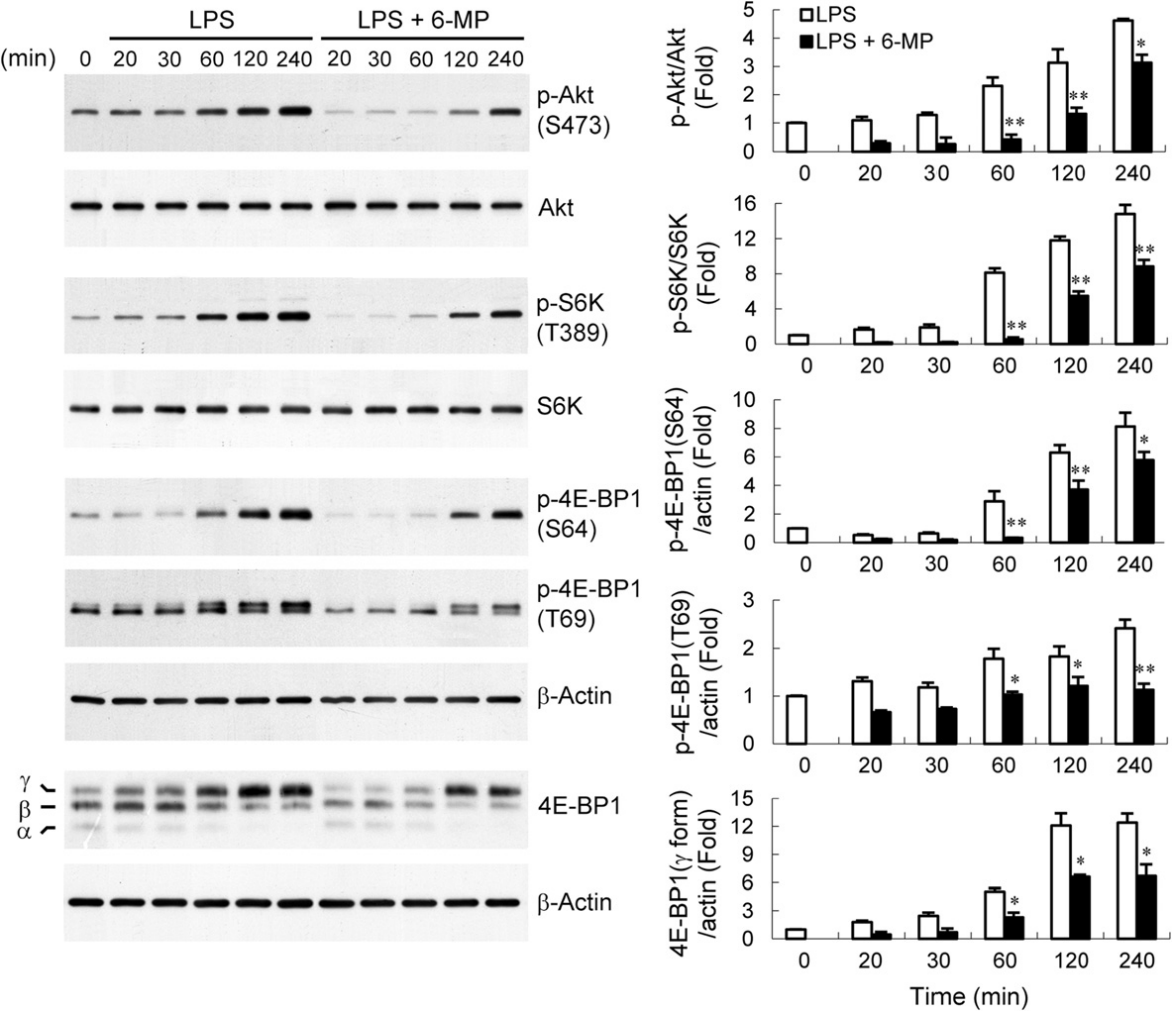
6-MP blocks PI3K/Akt/mTOR signaling. BV-2 cells were pretreated with vehicle or 6-MP (50 μM) for 4 h and then stimulated with LPS (100 ng/ml) for the indicated times. Western analysis was used to determine total and phosphorylated Akt, S6K and 4E-BP1 proteins in whole cell extracts. Data are presented as mean ± SEM for three independent experiments. *p < 0.05; **p < 0.01 compared with respective LPS alone

**Fig. 9:**
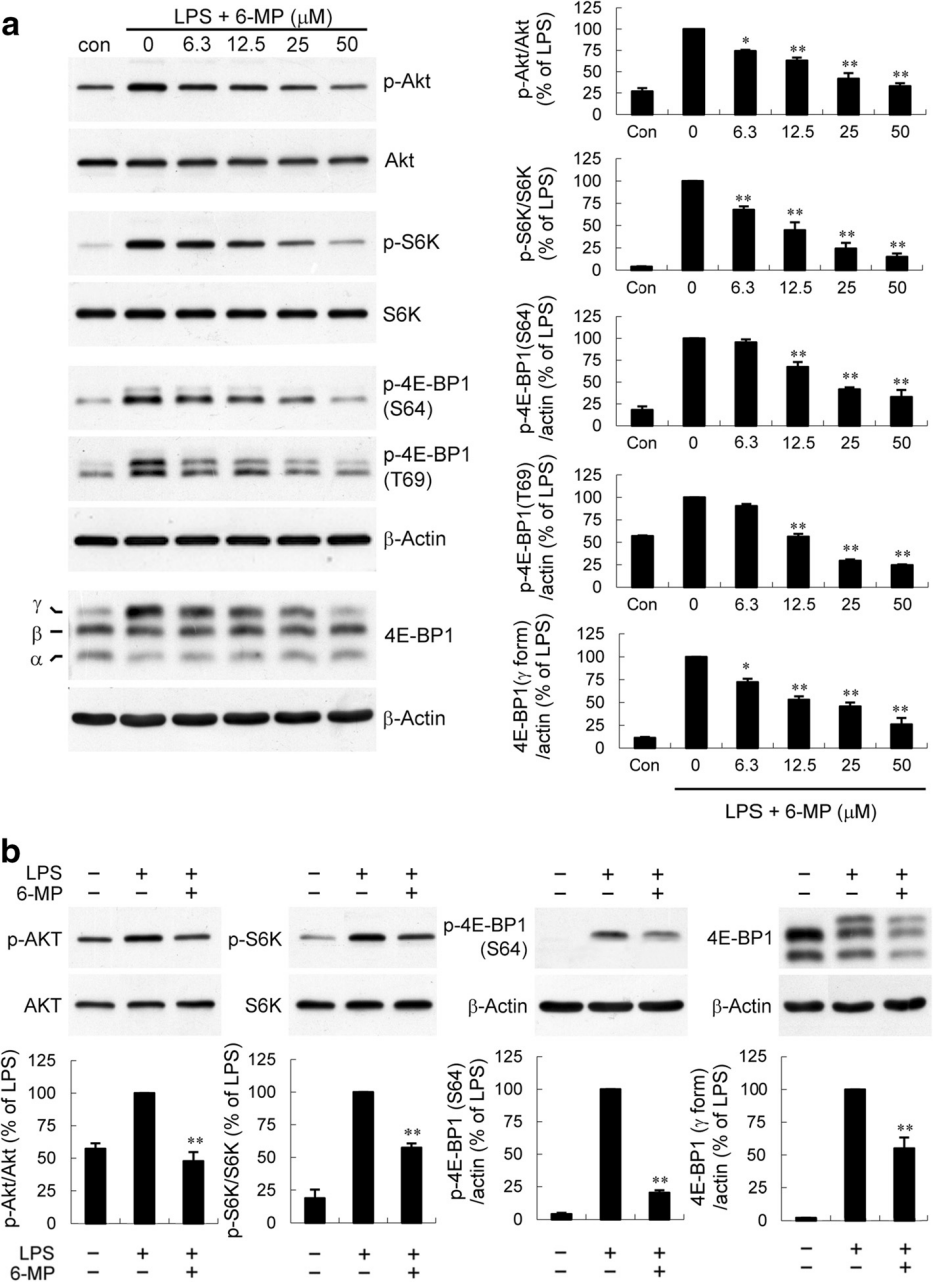
Dose-dependently inhibitory effect of 6-MP on activation of PI3K/Akt/mTOR signaling cascades. BV-2 cells (**a**) or primary microglia (**b**) were pretreated with vehicle or various concentrations (**a**) or 50 μM (**b**) of 6-MP for 4 h followed by exposure to LPS (100 ng/ml) for 60 min. Western analysis was performed as described in Fig. 8. Data are presented as mean ± SEM of three independent experiments. *p < 0.05; **p < 0.01 compared with LPS alone

The post-transcriptional regulation involving translational efficiency appears to play a critical role in the regulation of TNF-α expression [59, 60]. As mentioned above, the reduction in TNF-α mRNA levels caused by 6-MP was not proportional to the reduction in protein levels (Fig. 1b, c), suggesting that a post-transcriptional regulation is involved in the inhibitory effect of 6-MP. In addition, pretreatment with 6-MP (50 μM) for 4 h did not markedly decrease the levels of TNF-α mRNA, whereas the protein levels that were significantly reduced further supported this possibility. The signaling pathway, PI3-kinase (PI3K), is well known to control translation, involving signaling by Akt/protein kinase B and the mammalian target of rapamycin (mTOR) [61]. Activation of mTOR complex 1 (mTORC1) initiates the protein synthesis machinery via the phosphorylation of downstream targets p70 ribosomal protein S6 kinase (S6K) and the eukaryotic initiation factor 4E binding protein 1 (4E-BP1) [62, 63]. To test whether the PI3K pathway is regulated by 6-MP, we examined the phosphorylation of Akt, a well known target of PI3K. LPS stimulation resulted in a time-dependent phosphorylation of Akt (Fig. 8). Pretreatment of BV-2 cells with 6-MP markedly suppressed Akt phosphorylation caused by LPS. S6K is a common downstream effector of mTORC1 and plays a direct role in regulating translation [64]. Furthermore, activation of mTORC1 also results in phosphorylation of 4E-BP1 at Thr36, Thr45, Ser64, and Thr69 sites and the release of 4E-BP1 from eukaryotic initiation factor (eIF) 4E [65, 66], which is a limiting step in translation initiation. Therefore, we sought to determine whether 6-MP also regulates LPS-induced S6K and 4E-BP1 phosphorylation. Fig. 8 shows that LPS induced a time-dependent phosphorylation of S6K and 4E-BP1, which correlated with the kinetics of Akt activation by LPS. 6-MP pretreatment led to suppression of LPS-induced S6K phosphorylation and retention of 4E-BP1 in its active (hypophosphorylated) state. The γ-form is positive for phospho-Ser64 and contains all phosphorylated sites representing the hyperphosphorylated state. 4E-BP1 dissociation from eIF4E is generally associated with γ-form [67, 68]. We found that 6-MP also prevented the γ-form of 4E-BP1 accumulation caused by LPS (Fig. 8). Moreover, the investigation of the dose-response relationship for 6-MP-mediated blockade of PI3K-Akt-mTOR signaling pathway was performed over concentrations ranging from 6.3 to 50 μM. As illustrated in Fig. 9a, pretreatment of BV-2 cells with 6-MP led to a dose-dependent decrease in LPS-induced phosphorylation of Akt, S6K, and 4E-BP1 and accumulation of γ form of 4E-BP1. The 6-MP concentration required to achieve significant inhibition started between 6.3 and 12.5 μM, and the effect was more conspicuous at 50 μM. Similar findings were observed with LPS-stimulated primary microglia, where 6-MP pretreatment inhibited PI3K/Akt/mTOR signaling (Fig. 9b).

**Fig. 10:**
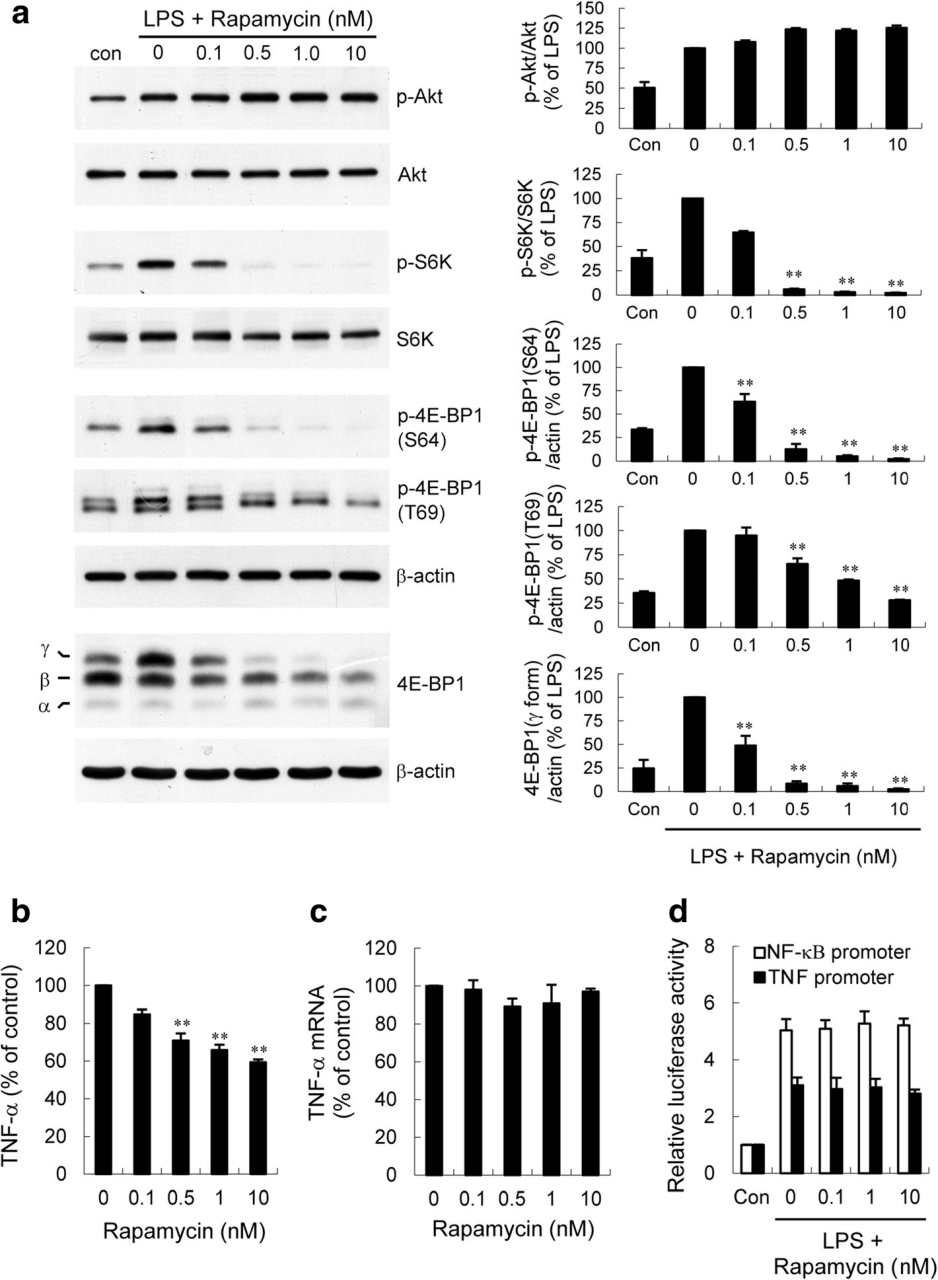
The mTOR inhibitor rapamycin reduces LPS-induced-TNF-α production through preventing mTOR signaling-mediated mRNA translation. BV-2 cells were pretreated with vehicle or various concentrations of rapamycin for 30 min followed by treatment with 100 ng/ml LPS for 60 min (western blotting), 2 h (TNF-α mRNA), or 6 h (TNF-α protein). Western analysis (**a**) was performed as described in Fig. 8. Released TNF-α (**b**) was measured by ELISA. The expression of TNF-α mRNA (**c**) was quantified by real-time RT-PCR. Data are presented as mean ± SEM of three independent experiments. **p < 0.01 compared with LPS alone. The level of TNF-α in cells treated with LPS alone was 18.1 ± 2.3 ng/ml. **d**. LPS-induced NF-κB or TNF-α promoter activity is not altered by rapamycin. Following transfection of BV-2 cells with NF-κB or TNF-α promoter luciferase construct, cells were pretreated with various concentrations of rapamycin for 30 min prior to treatment with LPS (100 ng/ml) for 6 h. Luciferase activity is presented as a fold of control. Data are presented as mean ± SEM of three independent experiments

In LPS-stimulated microglia, inactivation of Akt using a PI3K inhibitor has been shown to attenuate TNF-α production by preventing translation of mRNA [28]. We next used the mTOR inhibitor rapamycin to investigate the role of mTOR in the 6-MP-mediated decrease in TNF-α production. LPS-induced S6K phosphorylation in BV-2 cells was markedly abolished by rapamycin (Fig. 10a). In addition, rapamycin also blocked LPS-induced phosphorylation of 4E-BP1, leading to attenuation of the γ form of 4E-BP1 accumulation (Fig. 10a). Furthermore, LPS-induced TNF-α secretion was inhibited by rapamycin in a dose-dependent manner (Fig. 10b), which correlates with the decreased level of phosphorylated S6K and the γ form of 4E-BP1. However, rapamycin treatment had no inhibitory effect on LPS-induced TNF-α mRNA expression and TNF-α/consensus NF-κB promoter activity (Fig. 10c, d). These results suggest that downregulation of PI3K/Akt/mTOR signaling might contribute, at least partially, to the 6-MP-mediated decrease in TNF-α production after LPS exposure.

## Discussion

In chronic neurodegenerative diseases, microglial activation has been demonstrated to be an early sign that often precedes and triggers neuronal death [1–3]. Therefore, downregulation of microglia-mediated inflammation may offer prospective clinical therapeutic benefits for neuroinflammation-related neurodegenerative disorders. 6-MP, derived from the prodrug azathioprine, is applied as an immunosuppressive drug to treat systemic lupus erythematosus and inflammatory bowel diseases, such as Crohn’s disease [18]. We herein speculated that the 6-MP-mediated anti-inflammatory effects might not be limited in the periphery. Indeed, we found that microglial inflammatory responses could be downregulated through decreasing TNF-α secretion in LPS-stimulated microglia by 6-MP. In line with our results, 6-MP has been shown to robustly suppress MCP-1 expression in primary macrophages stimulated with LPS [26]. Using oxidized low-density lipoprotein (oxLDL)-treated RAW264.7 macrophage, Shao et al. [69] reported that MCP-1 and TNF-α mRNA expression are inhibited by 6-MP. Furthermore, 6-MP also exhibits an anti-inflammatory effect on endothelial cells [22, 70]. Our findings underscore the importance of 6-MP in regulation of inflammation and extend the role of 6-MP to a modulator of microglial activation.

Blockade of gene transcription in stimulated inflammatory cells is often due to one or multiple interruptions in the signaling transduction from the stimuli to the corresponding transcriptional cytokines. MAPKs are known to play a critical role in cytokine production [30, 71]. To understand the molecular mechanisms underlying the inhibitory effect of 6-MP, we studied the effect of 6-MP on LPS-induced activation of MAPKs. We found that 6-MP pretreatment did not inhibit LPS-induced phosphorylation of all three MAPKs. The major mechanism of action of 6-MP is believed to be inhibition of purine synthesis, and this effect is observed at a relatively high dose of 6-MP (500 μM) [72]. Other described mechanisms of action of 6-MP include inhibition of specific GTP-dependent proteins Rac1 and Rac2 in CD4^+^ T cells, which leads to blockade of T cell activation [21]. In addition, recent studies show that 6-MP inhibits Rac1 activation and thus subsequently attenuates the JNK signaling cascade, thereby decreasing inflammation in TNF-α-treated endothelial cells [22, 70]. Furthermore, 6-MP also has Rac1/JNK-dependent anti-inflammatory effect in macrophages exposed to interferon γ (IFN-γ) [23]. Our study found no reduction in JNK phosphorylation in microglia pretreated with 6-MP. Possible interpretations of our findings are that 6-MP inhibits phosphorylation of JNK in a cell type- and stimulus-specific manner.

The activation of Rac1 is inhibited by 6-MP, but with limited specificity, because not all immunosuppressive effects of 6-MP involve Rac1 inhibition [22, 23, 70]. 6-MP has been identified as an activator of orphan nuclear receptor Nur77 to increase the protein level and the transactivation function in several cell types [25, 49, 73]. Nur77 expression can be rapidly induced in monocytes and macrophages by a variety of inflammatory stimuli, such as TNF-α, LPS, and IFN-γ [52, 74]. The anti-inflammatory property of Nur77 has been demonstrated in various cell and animal models. In macrophages, overexpression of Nur77 reduces the expression of several inflammatory cytokines in response to LPS, TNF-α, and oxLDL [52, 69]. Moreover, Nur77 elevation suppresses TNF-α- and IL-1β-induced ICAM-1 and VCAM-1 expression in endothelial cells [53]. Mice deficient in Nur77 shows enhanced atherosclerosis, 2,4,6-trinitrobenzene sulfonic acid-induced colitis, and OVA-induced allergic airway inflammation [54–56]. Therefore, Nur77 may act as a molecular target for modulating of inflammation and Nur77 agonists may provide an effective treatment for inflammatory diseases. Herein, we found that the expression of Nur77 mRNA and protein were increased following 6-MP treatment in microglial cells. Furthermore, knockdown of Nur77 was shown to relieve the 6-MP-mediated inhibitory effect on LPS-induced TNF-α release. This is in agreement with a previous study reporting that Nur77 activation is responsible for 6-MP-mediated decrease in MCP-1 and TNF-α mRNA expression in oxLDL-stimulated macrophages [69]. Our findings suggest that in microglial cells, 6-MP mediates its anti-inflammatory function through, at least partially, upregulation of Nur77.

NF-κB and MAPKs play central roles in the regulation of proinflammatory cytokines expression [30, 31, 33, 71]. IκB masks p65 in the cytosol to prevent NF-κB-associated transcription in the nucleus. IκB is ubiquitinated for degradation upon phosphorylation by IKKs, which activates NF-κB. It has been proposed that Nur77 modulates inflammatory gene expression at least in part through transrepression of NF-κB in multiple cell systems [53–58]. In human umbilical vein endothelial cells, elevated expression of Nur77 results in the increase of IκBα expression and attenuation of TNF-α-induced translocation of p65 [53]. Knockdown of Nur77 enhances phosphorylation of IκBα in TNF-α-stimulated NCI-H292 lung epithelial cells [56]. However, these actions seem to be cell line and stimuli dependent. Furthermore, another mechanism was reported that NF-κB activity is downregulated through a direct Nur77 interaction with p65 to block its binding to the κB element [57, 58]. Very recently, Calvayrac et al. [51] have shown that NOR-1 overexpression prevents LPS-induced MAPKs activation in human vascular smooth muscle cells. 6-MP downregulated the expression of TNF-α mRNA in microglial cells upon LPS stimulation; however, the activation of MAPKs and the IκBα phosphorylation and degradation as well as the nuclear translocation did not seem contingent on 6-MP effects. The association of p65 with Nur77 was enhanced by 6-MP, whereas the difference in p65 binding to its response element in vitro was not observed between control cells and cells pretreated with 6-MP. Possible interpretation of our findings is that the increased amount of Nur77-p65 complex might be not yet enough to directly alter the DNA binding in our experimental conditions. Although the early steps leading to NF-κB activation were unaffected by 6-MP, our results showed that upregulated Nur77 was contributed to 6-MP-mediated inhibition of p65-dependent transcription. These findings suggest that 6-MP-induced Nur77 regulates NF-κB in LPS-stimulated microglia through reduction of transactivation activity of p65.

Once activated, NF-κB transcriptional activity is further regulated by inducible post-translational modifications, including phosphorylation and acetylation [33, 45]. A number of different phosphorylation sites have been identified on the p65 subunit. This phosphorylation is essential for NF-κB nuclear transportation, subunit dimerization, DNA binding, and finer regulation of NF-κB transcriptional activity [37–39]. Furthermore, prior studies implicated CBP/p300 as a critical regulator of NF-κB activity and showed that recruitment of CBP was enhanced by phosphorylation of p65 at Ser276 [37, 75]. PKA has been shown to be associated with the phosphorylation of p65 at Ser276 in response to LPS in murine 70Z/3 pre-B cells [40]. In TNF-α- or IL-1β-treated human HEK293 and murine L929sA cells, MSK1 is responsible for this phosphorylation [42, 43]. Despite the fact that neither PKA nor MSK1 activation seemed to be abolished by 6-MP, our data showed that 6-MP inhibited LPS-induced phosphorylation of p65 at Ser276 and decreased binding of p300 at the TNF-α promoter. A previous study showed that PKA is not to be a substantial factor for phosphorylation of this site in LPS-stimulated RAW264.7 macrophages [76]. Whether LPS-induced p65 phosphorylation at Ser276 in microglia involves PKA activation needs further investigation. These findings lead us to deduce that other kinases (as yet unidentified) may be involved in Ser276 phosphorylation in LPS-stimulated microglia, which could be influenced by 6-MP. Blockade of 6-MP-mediated repression by knockdown of Nur77 restored LPS-induced p65 phosphorylation at Ser276, whereas the reduced occupancy of p300 at the TNF-α promoter was not reversed. These results suggest that in microglia, phosphorylation of p65 at Ser276 might be necessary but not sufficient for p300 recruitment to TNF-α promoter. Indeed, post-translational modifications of CBP/p300, such as methylation, have also been shown to influence its binding to transcription factors [77, 78]. Site-specific acetylation of p65 regulates discrete biological actions of the NF-κB complex [44, 45]. Acetylation of lysine 310 has been shown to be required for full transcriptional activity of p65. In the nucleus, p65 associates with p300/CBP transcriptional co-activators. It appears that the major effect of CBP/p300 on NF-κB-dependent transcription is via acetylation of proteins and histones in the transcriptional apparatus and chromatin remodeling [45, 48]. Nur77 has been found to interact with p300 and negatively regulate its histone acetyltransferase activity, resulting in suppression of the acetylation and transcriptional activity of many p300-regulated transcription factors, e.g., NF-κB p65 [79]. Furthermore, the p300-induced histone H3 acetylation is also repressed by the presence of Nur77, suggesting that Nur77 would affect general transcription machinery through chromatin remodeling [79]. In the present study, the addition of 6-MP decreased levels of acetylated p65 and reduced accumulation of Ac-H3 at the TNF-α promoter following LPS stimulation and downregulation of Nur77 reversed this inhibitory effect. Taken together, our data suggest that 6-MP suppresses TNF-α expression through, at least in part, Nur77-mediated downregulation of NF-κB transcriptional efficiency by inhibiting p65 phosphorylation and acetylation. Moreover, upregulated Nur77 is also involved in 6-MP-mediated decrease of acetylated histone H3 at TNF-α promoter, ultimately leading to compacting chromatin structures and impairing binding of p65 to induce TNF-α gene expression.

It is known that the post-transcriptional regulation involving translational efficiency contributes to the modulation of TNF-α expression [59, 60]. Our study showed that 6-MP-mediated reduction of TNF-α mRNA levels is not proportional to the protein production. Therefore, these results suggest that 6-MP’s inhibitory effect on TNF-α production may involve a translational event. LPS-induced activation of MAPK is shown to influence TNF-α gene expression at the level of translation [60, 80]. The data presented in this study showing that LPS-induced activation of MAPK remain unaltered following 6-MP pretreatment. We therefore tend to speculate that another mechanism controlling the TNF-α mRNA translation might be involved in 6-MP-mediated reduction of TNF-α protein. The PI3K/Akt/mTOR signaling pathway has been shown to play an important role in the modulation of translation [61, 65, 66]. 4E-BP1 is a direct target of mTORC1, which is known to control 4E-BP1 activity through hyperphosphorylation of this protein [65, 66]. 4E-BP1 phosphorylation is an important step in controlling the rate of initiation of translation in mammalian cells [61]. Phosphorylation of 4E-BP1 dissociates it from eIF4E, relieving the translational inhibition. Moreover, S6K is another downstream effector of mTORC1 and also plays a direct role in regulating translation [64]. In this report, we found that pretreatment with 6-MP resulted in attenuation of LPS-induced Akt phosphorylation and inhibition of mTORC1 activity, which was confirmed by a significant decrease in 4E-BP1 and S6K phosphorylation. Additionally, inhibition of mTOR activity by rapamycin was shown to reduce the production of TNF-α protein but had no effect on mRNA levels. These data are consistent with a recent report showing that in LPS-stimulated macrophages, a second-generation mTOR kinase inhibitor INK128 suppresses TNF-α production by downregulating TNF-α biosynthesis [81]. The present results suggest that the inhibitory effects of 6-MP on TNF-α production are also acted at translational level by blocking PI3K/Akt/mTOR signaling.

## Conclusions

**Fig. 11:**
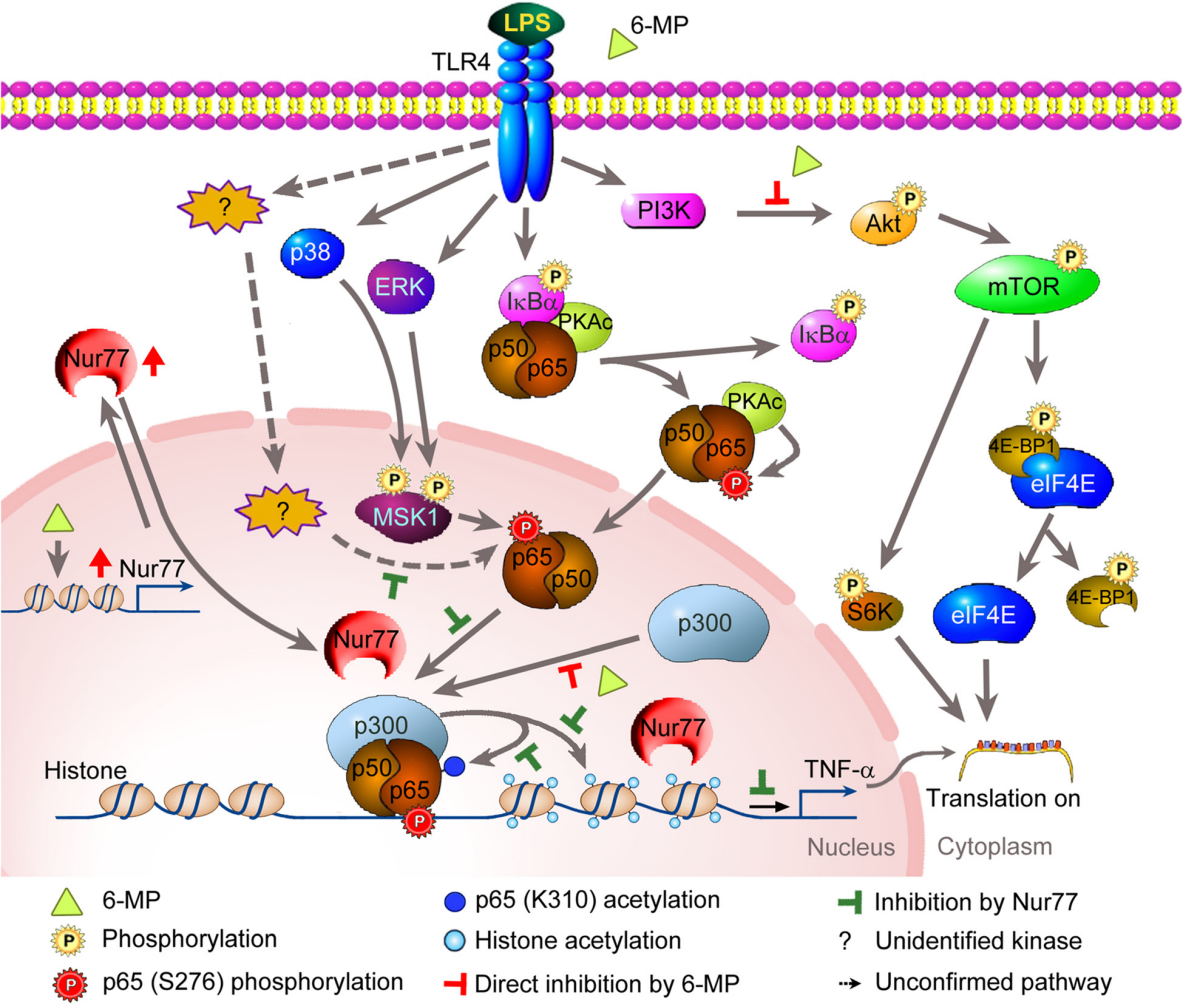
Proposed model for 6-MP inhibition of LPS-induced TNF-α expression. Release of NF-κB dimmers following LPS stimulation is linked to phosphorylation of p65 at Ser276, either in the cytoplasm by PKAc or in the nucleus by MSK1. In microglia, 6-MP upregulates Nur77 expression. Nur77 subsequently attenuates p65 phosphorylation (S276) by inhibiting a yet unidentified kinase and acetylation (K310) by dampening p300 activity to limit transcriptional activation of p65. Furthermore, Nur77 blocks the acetylation of histone H3 at TNF-α promoter, ultimately leading to compacting chromatin structures and impairing binding of p65. Both events reduce the LPS-induced TNF-α gene transcription. On the other hand, the inhibitory action of 6-MP also occurs via inactivation of PI3K/Akt/mTOR signaling to downregulate translation

In summary, a schematic overview of the mechanisms by which 6-MP inhibits TNF-α expression is presented in Fig. 11. 6-MP dampens TNF-α gene expression in LPS-stimulated microglia via Nur77-mediated impairment of p65 phosphorylation and acetylation and an epigenetic regulation mechanism. Moreover, blockade of PI3K/Akt/mTOR signaling-mediated translation of TNF-α mRNA is also involved in 6-MP’s inhibitory actions. Because downregulation of microglia-mediated inflammation may offer prospective clinical therapeutic benefits for neuroinflammation-related neurodegenerative disorders, the findings presented here may provide new insight into the clinical applications of this old drug in targeting various neurodegenerative diseases.

## Additional files

Additional file 1: Figure S1. LPS-induced activation of PKA and MSK1 are not suppressed by 6-MP. BV-2 cells were stimulated with 100 ng/ml LPS for the indicated times with or without 16 h pretreatment with 6-MP (50 μM). Immunoblots were set up to detect phosphorylated VASP (Ser157) and MSK1 (Ser376). Images are representative of three independent experiments. (TIF 400 kb) Additional file 2: Figure S2. 6-MP does not increase Nurr1 or NOR-1 protein levels in BV-2 cells. Cells were treated with various concentrations of 6-MP for 1 h (mRNA) or 16 h (protein). Nurr1 (a) and NOR-1 (b) transcripts and proteins were analyzed using real-time RT-PCR or Western blotting, respectively. (TIF 332 kb) Additional file 3: Figure S3. Physical interaction between Nur77 and p65. BV-2 cells were pretreated with 6-MP (50 μM) for 16 h followed by exposure to LPS (100 ng/ml) for 60 min. Nuclear extracts were harvested for immunoprecipitation (IP) experiments using anti-Nur77 and anti-p65 antibodies. Immunoblot (IB) analyses of the immunoprecipitates were performed using these antibodies. The immunoblots are representative of three independent experiments. (TIF 280 kb)
