# Supplementary figures and images for: 6-Mercaptopurine attenuates tumor necrosis factor-α production in microglia through Nur77-mediated transrepression and PI3K/Akt/mTOR signaling-mediated translational regulation
Source: J Neuroinflammation. 2016 Apr 13;13:78. doi: 10.1186/s12974-016-0543-5 (PMC4831152; doi:10.1186/s12974-016-0543-5)

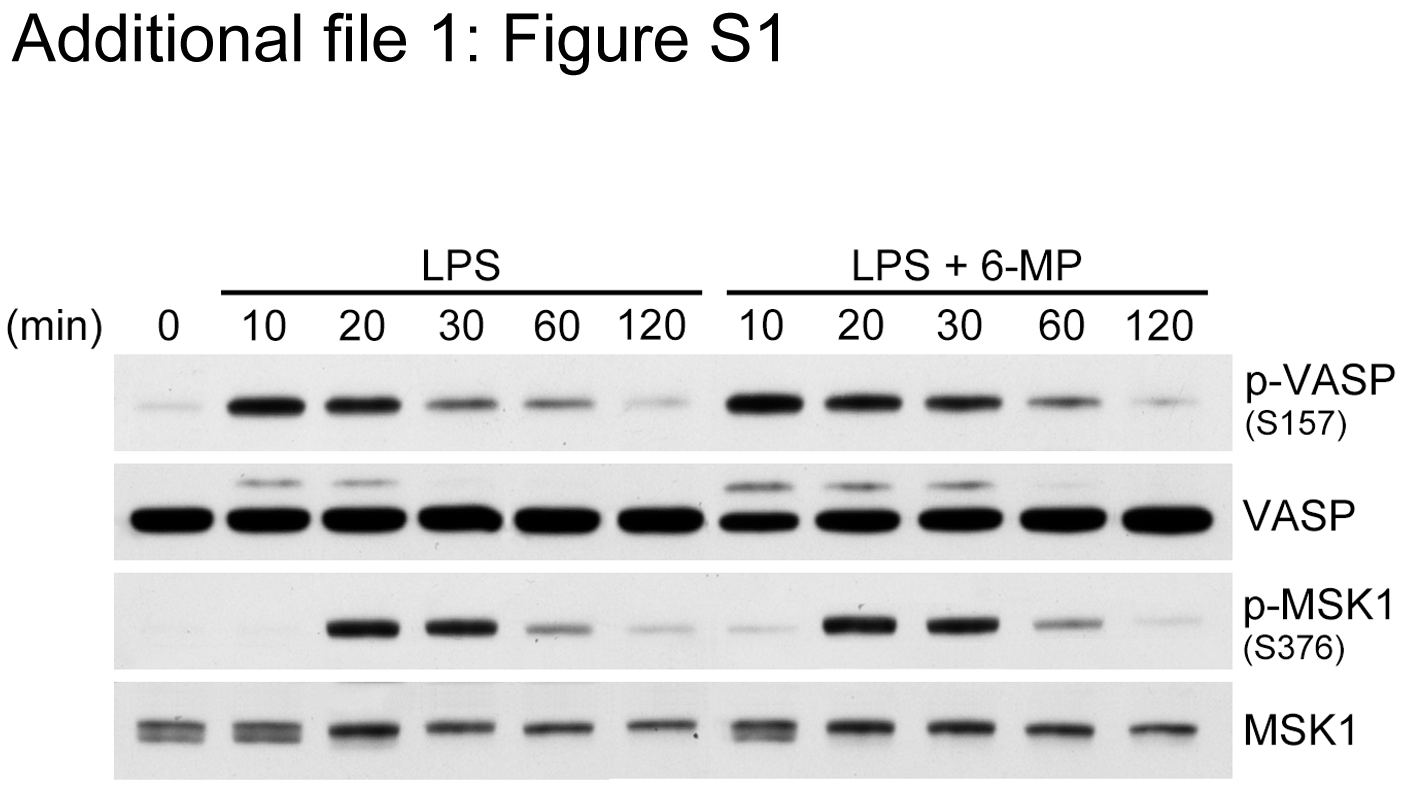

Supplement: Additional file 1: Figure S1. — LPS-induced activation of PKA and MSK1 are not suppressed by 6-MP. BV-2 cells were stimulated with 100 ng/ml LPS for the indicated times with or without 16 h pretreatment with 6-MP (50 μM). Immunoblots were set up to detect phosphorylated VASP (Ser157) and MSK1 (Ser376). Images are representative of three independent experiments. (TIF 400 kb) [file 12974_2016_543_MOESM1_ESM.tif]

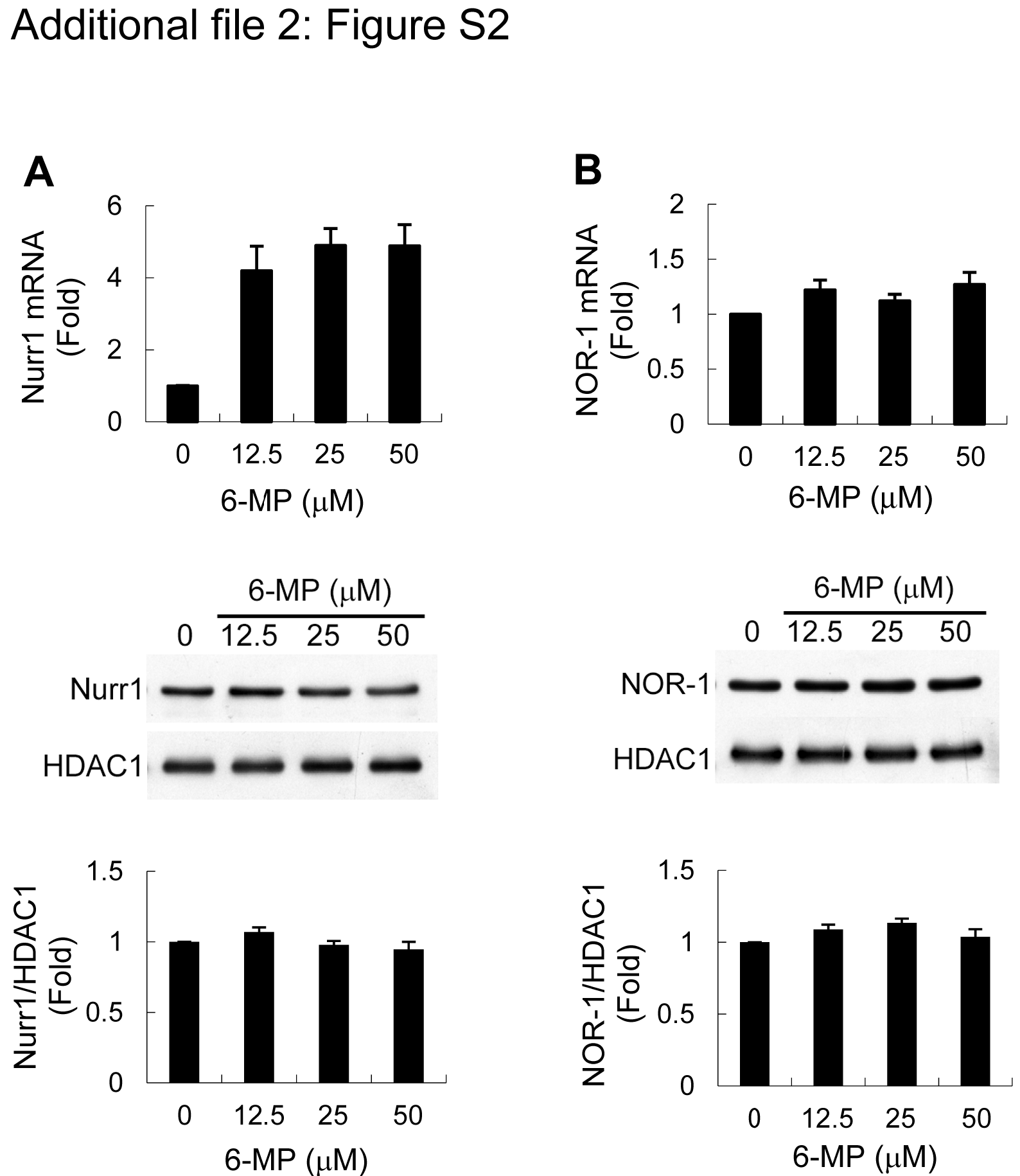

Supplement: Additional file 2: Figure S2. — 6-MP does not increase Nurr1 or NOR-1 protein levels in BV-2 cells. Cells were treated with various concentrations of 6-MP for 1 h (mRNA) or 16 h (protein). Nurr1 (a) and NOR-1 (b) transcripts and proteins were analyzed using real-time RT-PCR or Western blotting, respectively. (TIF 332 kb) [file 12974_2016_543_MOESM2_ESM.tif]

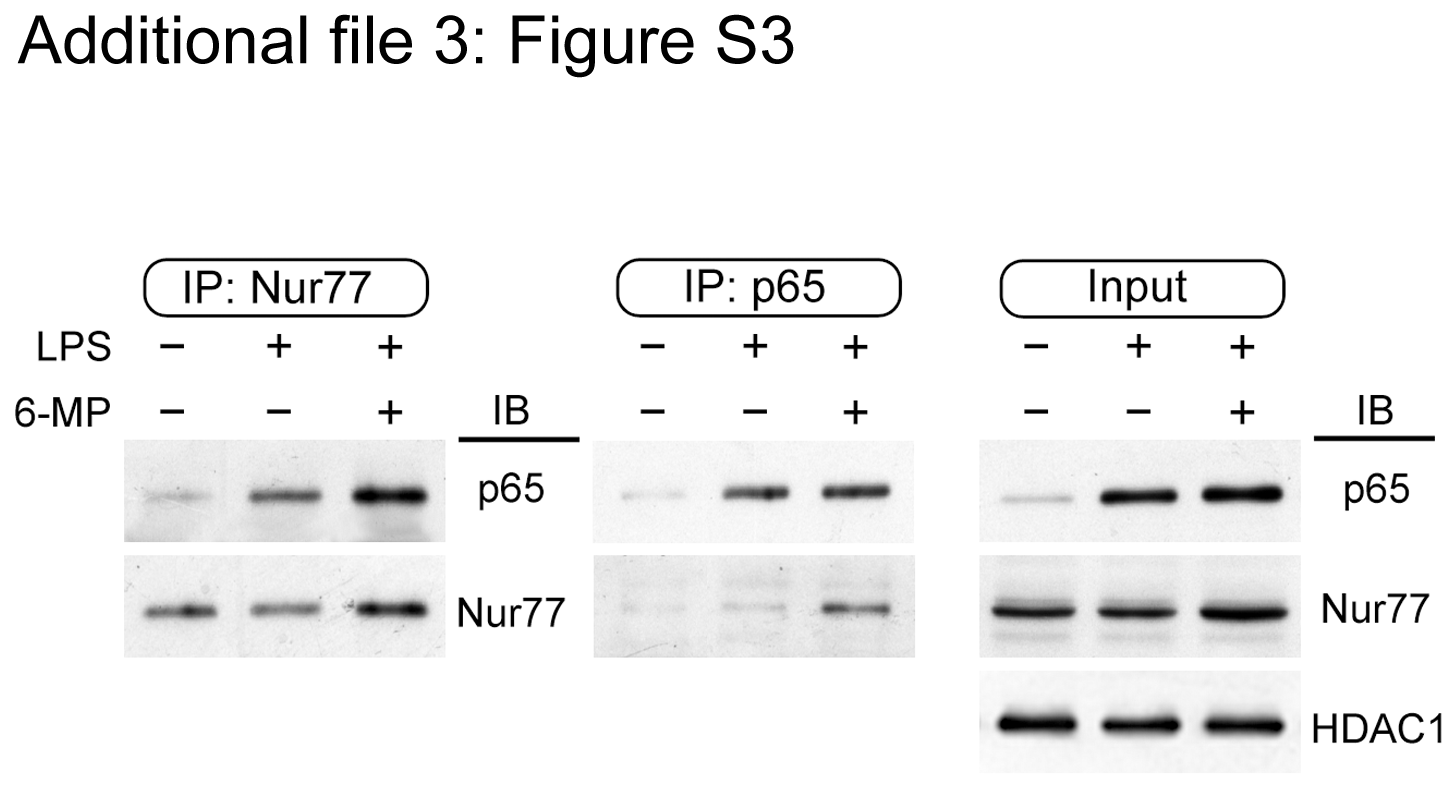

Supplement: Additional file 3: Figure S3. — Physical interaction between Nur77 and p65. BV-2 cells were pretreated with 6-MP (50 μM) for 16 h followed by exposure to LPS (100 ng/ml) for 60 min. Nuclear extracts were harvested for immunoprecipitation (IP) experiments using anti-Nur77 and anti-p65 antibodies. Immunoblot (IB) analyses of the immunoprecipitates were performed using these antibodies. The immunoblots are representative of three independent experiments. (TIF 280 kb) [file 12974_2016_543_MOESM3_ESM.tif]
